# Developmental changes in the reflectance spectra of temperate deciduous tree leaves and implications for thermal emissivity and leaf temperature

**DOI:** 10.1111/nph.16909

**Published:** 2020-11-29

**Authors:** Andrew D. Richardson, Donald M. Aubrecht, David Basler, Koen Hufkens, Christopher D. Muir, Leonard Hanssen

**Affiliations:** ^1^ Center for Ecosystem Science and Society Northern Arizona University Flagstaff AZ 86011 USA; ^2^ School of Informatics, Computing and Cyber Systems Northern Arizona University Flagstaff AZ 86011 USA; ^3^ Department of Organismic and Evolutionary Biology Harvard University Cambridge MA 02138 USA; ^4^ Department of Applied Ecology and Environmental Biology Ghent University Ghent Belgium; ^5^ INRA Aquitaine UMR ISPA Villenave d'Ornon France; ^6^ School of Life Sciences University of Hawai’i Mānoa Honolulu HI 96822 USA; ^7^ National Institute of Standards and Technology (NIST) Gaithersburg MD 20899 USA

**Keywords:** directional‐hemispherical reflectance (DHR), cuticle, Fourier transform infrared (FT‐IR), leaf development, leaf temperature, mid‐infrared (MIR), phenology, thermal remote sensing

## Abstract

- Leaf optical properties impact leaf energy balance and thus leaf temperature. The effect of leaf development on mid‐infrared (MIR) reflectance, and hence thermal emissivity, has not been investigated in detail.
- We measured a suite of morphological characteristics, as well as directional‐hemispherical reflectance from ultraviolet to thermal infrared wavelengths (250 nm to 20 µm) of leaves from five temperate deciduous tree species over the 8 wk following spring leaf emergence.
- By contrast to reflectance at shorter wavelengths, the shape and magnitude of MIR reflectance spectra changed markedly with development. MIR spectral differences among species became more pronounced and unique as leaves matured. Comparison of reflectance spectra of intact vs dried and ground leaves points to cuticular development – and not internal structural or biochemical changes – as the main driving factor. Accompanying the observed spectral changes was a drop in thermal emissivity from about 0.99 to 0.95 over the 8 wk following leaf emergence.
- Emissivity changes were not large enough to substantially influence leaf temperature, but they could potentially lead to a bias in radiometrically measured temperatures of up to 3 K. Our results also pointed to the potential for using MIR spectroscopy to better understand species‐level differences in cuticular development and composition.

## Introduction

Leaves are the primary interface between plants and the atmospheric environment, with which they exchange gases, momentum, heat and radiant energy (Jones, 2013). It has long been recognised that these exchanges determine leaf temperature (Brown & Wilson, 1905), which influences key physiological processes including photosynthesis, transpiration and cellular respiration (Brown & Escombe, 1905; Still *et al*., 2019).

Leaf structure and biochemical composition play important roles in regulating radiant fluxes by affecting the reflectance, absorptance and transmittance of electromagnetic radiation, particularly shortwave (Gates *et al*., 1965; Ollinger, 2011). The shortwave reflectance spectra of leaves has been observed to change with leaf development (Gates *et al*., 1965; Gausman *et al*., 1971), but much less well studied is how leaf development affects longwave properties, including thermal emissivity (*ε*). Emissivity is of particular importance because it is a physical control on: (i) how much longwave is absorbed vs reflected from the surrounding environment; and (ii) how much longwave is emitted by a leaf at a given temperature (Gates & Tantraporn, 1952; Fuchs & Tanner, 1966). Leaves with high *ε* (near unity) are more like an idealised blackbody and should be better able to reduce excess heat load through thermal energy dissipation, as has been proposed for desert plants (Arp & Phinney, 1980).

The increasing availability of thermal imaging sensors has resulted in renewed interest in measurements of leaf and canopy temperatures using radiometric approaches (Jones, 2004; Aubrecht *et al*., 2016; Still *et al*., 2019). These methods require knowledge of *ε*; if *ε* is not accurately known, then errors in radiometric temperature measurement are unavoidable (Buettner & Kern, 1965; Fuchs & Tanner, 1966). A 0.01 error in *ε* will cause an error of about 0.7 K (Arp & Phinney, 1980; Norman *et al*., 1990; Jones, 2004), with the estimated temperature below the true temperature if the value used for *ε* is above the true *ε* (i.e. assuming *ε* = 1.00 when actually *ε* = 0.99). Errors in *ε* could therefore easily lead to biases in radiometric leaf temperature measurements of 0.5 K or more. Such errors would be large enough to be physiologically meaningful in many studies.

Leaf *ε* has been quantified in a variety of ways. Many studies have used an ‘emissivity box’ (e.g. Buettner & Kern, 1965; Fuchs & Tanner, 1966; Arp & Phinney, 1980; Sutherland, 1986; Rubio *et al*., 1997) to determine *ε* based on simultaneous temperature and radiance measurements. Alternatively, *ε* can be calculated from mid‐infrared (MIR; defined as 3–50 µm according to ISO 20473) directional‐hemispherical reflectance (DHR) spectra following Kirchhoff's law of thermal radiation (Salisbury & D’Aria, 1992; Salisbury *et al*., 1994; Hecker *et al*., 2013). Kirchhoff’s law states that for an opaque surface, *ε* at wavelength λ equals 1 minus the DHR at that wavelength ($\varepsilon_{\lambda }=1‐R_{\lambda },$ Nicodemus, 1965). An average *ε* value across the 8–14 µm atmospheric window is typically used for biological and geological samples (Salisbury & D’Aria, 1992). The development of commercially available diffuse gold integrating spheres in the 1980s has greatly facilitated the measurement of MIR DHR spectra (Hanssen & Snail, 2001).

Early measurements of MIR leaf reflectance had limited resolution in terms of both spectral resolution and measurement precision (Gates & Tantraporn, 1952; Wong & Blevin, 1967). These data indicated that leaf reflectance across the MIR spectrum was generally very low, but improved measurement technology would be required for the underlying spectral richness to be appreciated. While leaves have not been found to exhibit the strong MIR reflectance features and high spectral contrast of mineral and soil samples (e.g. Sutherland, 1986), it is now quite clear that the MIR reflectance spectra are far from featureless (Salisbury, 1986; Ribeiro da Luz & Crowley, 2007). Indeed, leaves have distinct MIR spectral fingerprints that vary according to species and growth conditions, and which typically exhibit some narrow features that are strikingly different from the broader peaks and troughs common to the more familiar regions of the spectrum.

Across the electromagnetic spectrum, variation in leaf reflectance is driven by different leaf properties and traits (Gates *et al*., 1965; Gausman *et al*., 1971; Ollinger, 2011). Visible (VIS) wavelength (400–750 nm) reflectance is largely determined by leaf pigments. Within the near infrared (NIR; 750–1350 nm), reflectance depends on leaf structure, whereas in the shortwave infrared (1350–3000 nm) reflectance largely depends on leaf water content. At longer MIR wavelengths, organic and inorganic molecules have fundamental absorption features that are driven by the stretching and bending of chemical bonds as those bonds interact with radiation of a particular wavelength (Türker‐Kaya & Huck, 2017). However, MIR radiation does not penetrate deeply into most materials, usually only a few µm (Chalmers, 2013; Roberts *et al*., 2018). Thus, observed MIR features are driven by the structure and composition of the outermost tissue layers, specifically the cuticle and upper epidermal wall (Salisbury, 1986; Elvidge, 1988; Ribeiro da Luz, 2006; Ribeiro da Luz & Crowley, 2007).

The cuticle is a highly complex, composite biopolymer (Domínguez *et al*., 2017), comprised of a large number of organic compounds that vary in composition and arrangement depending on species (Holloway, 1994; Jeffree, 2007; Fernández *et al*., 2017). The cuticle determines the permeability and wettability of the leaf surface, and protects the underlying epidermal cells (Holloway, 1994). The majority of this layered membrane is comprised of a mixture of cutins and intracuticular waxes. The cuticle proper is typically topped with a thin layer of epicuticular wax. During leaf expansion and maturation, the mass (per unit area) and thickness of the cuticle may more than double, while the amount of soluble cuticular waxes can increase three‐fold (Viougeas *et al*., 1995). Full development of the cuticle may take 60 d or more in some species, with diverse epicuticular compounds (e.g. acetates, alcohols, and alkanes) being deposited at different stages of development (Jetter & Schäffer, 2001). Just as the MIR reflectance spectra vary across species because of differences in cuticular composition, there should be differences in MIR reflectance (and hence *ε*) associated with cuticular development. Thus, cuticular properties may influence the longwave radiation balance, and the thermal regime, of a leaf.

We conducted a study to evaluate changes in leaf reflectance associated with leaf development and maturation in the 8 wk following spring emergence. Many previous studies have quantified developmental changes in reflectance from 400 nm to 1100 nm (Gates *et al*., 1965; Gausman *et al*., 1971; Gamon & Surfus, 1999; Liu *et al*., 2009), and occasionally to 2500 nm (Yang *et al*., 2016; Chavana‐Bryant *et al*., 2017; Wu *et al*., 2017). By comparison, developmental changes in MIR DHR reflectance have received relatively little attention to date, with extremely limited analysis presented by Gates & Tantraporn (1952), Wong & Blevin (1967), and Ribeiro da Luz (2006). None of these previous studies looked at changes in MIR reflectance during leaf expansion. This lack of attention is despite the potential importance of MIR reflectance in the context of leaf energy balance, actual leaf temperature and radiometrically measured leaf temperature.

We tracked leaf development of five temperate deciduous species and regularly measured reflectance across the spectrum from ultraviolet (UV) to thermal bands (250 nm to 20 µm). Specifically, we asked:

1. How does leaf reflectance vary across species, and in what spectral regions are these differences most pronounced?
2. How does leaf reflectance vary with development, that is over the 8 wk following leaf emergence, and does this affect *ε*?
3. Are developmental changes in *ε* large enough to influence either leaf temperature itself, or radiometric measurement of leaf temperature?

## Materials and Methods

### Sample collection protocol

Our study focused on the foliage of five temperate deciduous tree species: paper birch (*Betula papyrifera* Marsh.), American beech (*Fagus grandifolia* Ehrh.), red maple (*Acer rubrum* L.), northern red oak (*Quercus rubra* L.), and quaking aspen (*Populus tremuloides* Michx.). These species were selected to be representative of the eastern deciduous forest of North America.

We collected samples from trees growing in the wild along a forest edge (outer canopy, or sun leaf, branches) in Intervale, New Hampshire, USA (44.0917°N, 71.1520°W, 160 m elevation). Leaf‐out occurred during the last week of April for birch, maple and aspen, during the first week of May for beech, and during the second week of May for oak. Once leaves had grown to sufficient size (2 cm minimum dimension) to fully cover the sample port on the Pike integrating sphere, we collected samples weekly during May, and then biweekly through the end of June. By this time, leaf elongation and maturation were expected to be complete, based on previous phenological studies (Richardson & O’Keefe, 2009; Keenan *et al*., 2014).

On each sampling date, we clipped short branchlets, *c*. 20 cm in length and containing 4–8 leaves, from three different individuals of the same species. Following Richardson & Berlyn (2002) and Ribeiro da Luz (2006), we wrapped samples in moist paper towel and kept them cool and dark in clean plastic zip‐top bags until measurements were completed the following day.

Leaf morphology, anatomy and biochemical composition are known to vary along the canopy light gradient, that is between sun and shade leaves (Jackson, 1967; Lichtenthaler *et al*., 1981). Sun leaves typically have a thicker cuticle than shade leaves (Osborn & Taylor, 1990; Ashton & Berlyn, 1994). To complement our study of the developmental changes in leaf reflectance spectra, we collected a separate set of samples at the end of June to investigate differences in reflectance between sun and shade leaves (Gausman, 1984). Shade leaf samples were collected from shaded lower canopy branches, or individuals growing in the shaded understory, for an additional three trees of each species.

### Measurement protocols

Samples were collected in the field during late afternoon, transported to the laboratory and promptly analysed the following morning. We conducted all measurements for one species before moving on to the next species. Leaves were kept in zip‐top bags except when measurements were being made. For each species, a full set of measurements took about 20 min.

#### UV/VIS/NIR reflectance spectra

We measured leaf reflectance from 250 to 2500 nm using a Lambda 750S UV/VIS/NIR spectrophotometer (PerkinElmer Life and Analytical Sciences, Shelton, CT, USA). This instrument features a double‐beam double‐monochromator design, meaning that the reference and sample beams were measured simultaneously with each scan. We used a 100 mm diameter integrating sphere with built‐in InGaAs (indium gallium arsenide) detector (PerkinElmer part no. L6020371). Samples were illuminated by twin deuterium and tungsten–halogen source lamps, and we used a wedge‐shaped sample holder for an 8° angle of incidence. The instrument was operated using PerkinElmer's UV WinLab software. Baseline scans were conducted with a Spectralon certified reflectance standard (Labsphere, Sutton, NH, USA). Raw data were saved in PerkinElmer’s binary.sp format, and processed reflectance spectra were exported at 5 nm as .csv text files. In subsequent analyses, we averaged the spectra to 10 nm.

#### NIR/MIR reflectance spectra

We measured leaf reflectance from 2 to 20 µm (500–5000 cm^−1^ wavenumbers) using a Nicolet iS10 (Thermo Scientific, Waltham, MA, USA) Fourier transform infrared (FT‐IR) spectrometer with a Mid‐IR IntegratIR 76.2 mm (3 inch) gold‐coated integrating sphere (Pike Technologies, Madison, WI, USA). The sphere featured an integrated mercury cadmium telluride (MCT) detector, which was cooled with liquid nitrogen. Power was delivered to the instrument through a line conditioner with automatic voltage regulation (Model LC1200; Tripp Lite, Chicago, IL, USA). From powering on the instrument to the completion of each measurement session, we purged the instrument with dry N_2_ gas to minimise artefacts associated with H_2_O and CO_2_ absorption features within the range of our measurements. We used the spectrometer’s built‐in MIR Ever‐glo source, with a 12° angle of incidence, for sample illumination.

The IntegratIR sphere is upward looking. Leaf samples were placed on the open port, and held in place with a circular washer larger than the 18 mm (¾ inch) port diameter. The sphere has a built‐in flipper mirror that enables measurements to be made using the comparison method. This has the advantage of eliminating changes in optical throughput associated with the substitution method (Hanssen & Snail, 2001). Briefly, with a sample on the open sample port, the background (reference) spectrum was collected first, with the mirror in the ‘reference’ position. This was then followed by a second spectrum, collected with the mirror in the ‘sample’ position.

Each measurement consisted of 64 scans, which were then averaged. Data collection and conversion of interferograms to spectra was conducted using Thermo Scientific's OMNIC software. Raw data were saved in Thermo Scientific's binary .spa format, and processed reflectance spectra were exported at 0.482 cm^−1^ as .csv text files. In subsequent analyses, data were aggregated to a spectral resolution of 4.82 cm^−1^, which corresponded to 0.0939 µm spacing at 14 µm, and 0.0014 µm spacing at 2 µm.

The procedures by which the measured reflectance spectra were converted to corrected sample reflectance measurements are described in Supporting Information Methods S1 (also described in Figs S1–S3; Tables S1–S3), where we also present a series of measurements which were used to quantify the expanded uncertainty for our measurements. These analyses showed that measurement uncertainty increased rapidly at wavelengths longer than about 14 µm: the expanded uncertainty (*k* = 2) of a low‐reflectance reference material (ESLI Velvet) was found to be roughly eight‐fold larger at 20 µm (4%) than the 0.5% we calculated for the 8–14 µm range. Hence, we generally restricted our analysis of FT‐IR data to wavelengths from 2 to 14 µm, although our initial presentation (to be described later in the Results section) of the spectra made use of the entire range from 2 to 20 µm.

Total reflectance from a surface consists of both diffuse (multiangular) and specular (mirror‐like) components, that is *R*_total_ = *R*_diffuse_ + *R*_specular_. Unless otherwise noted, the reflectance spectra presented here represented *R*_total_. However, the IntegratIR sphere we used featured a specular exclusion port that, when open, resulted in the specular component being directed out of the sphere, so that only the diffuse component was measured by the detector. For the sun and shade leaves collected on the final sampling date, we measured both the total reflectance and the diffuse component and calculated the specular component by difference. Additional details are described in Methods S2 (described in Fig. S4).

### Spectral indices

From the UV/VIS/NIR and MIR reflectance spectra we calculated a variety of spectral indices to broadly characterise leaf optical properties. Using red edge reflectance at 705 nm (*R*_705_), and NIR plateau reflectance at 750 nm (*R*_750_), we calculated the chlorophyll normalised difference index (Chl NDI) as:

$$\text{Chl NDI}=\frac{R_{750}‐R_{705}}{R_{750}+R_{705}}$$

The Chl NDI has been shown to be linearly correlated with chlorophyll content on an area basis (mg Chl m^−2^ leaf area) (Gitelson & Merzlyak, 1994; Richardson *et al*., 2002).

We calculated the mean shortwave reflectance (*R*_SW_) by weighting the measured 250–2500 nm reflectance spectra by the solar (direct + circumsolar) irradiance spectrum (ASTM G173‐03 Reference Spectra Derived from Smarts v.2.9.2; Gueymard, 2004). Mean shortwave reflectance is a leaf‐level approximation to albedo (Bartlett *et al*., 2011).

We calculated leaf emissivity, *ε*, following Kirchhoff's law. We used 8–14 µm for our definition of the thermal band, following Ribeiro da Luz & Crowley (2007).

### Leaf area and colour

At the conclusion of spectral measurements for each sampling date, we scanned eight leaves of each species at 300 dpi on a digital flatbed scanner (Perfection 3170 Photo Scanner; Epson America Inc., Long Beach, CA, USA). Three‐colour images (red, green and blue) were saved in minimally compressed .jpg format. The images were then processed in R to determine the mean one‐sided surface area of each leaf (*A*_leaf_, cm^2^) and the mean leaf colour signature, which we characterised as an RGB DN triplet (mean red DN, mean green DN, mean blue DN), where DN denotes an 8 bit digital number for each channel (binary integer). We calculated the green chromatic coordinate as *G*_cc_ = (green DN)/(red DN + green DN + blue DN), and similarly the red and blue chromatic coordinates, *R*_CC_ and *B*_CC_.

For quality control, a green reference (B32 paint swatch; Ace Hardware, Oak Brook, IL, USA) was included in each scan (Keenan *et al*., 2014). The colour of this standard (mean ± 1 standard deviation (SD)) was highly consistent (red DN: 167.9 ± 0.6; green DN: 198 ± 0.7; blue DN: 160 ± 0.7) over the course of this study.

### Leaf dry matter and water content

For the same set of eight leaves, we also determined mean fresh leaf mass (*M*_fresh_, g) on a three‐decimal balance (Adventurer Pro AV53; Ohaus Corp., Parsippany NJ, USA). We then oven dried (60 °C) the leaves to constant weight to determine mean dry leaf mass (*M*_dry_, g). From these and the *A*_leaf_ data, we calculated the fresh (g m^−2^) mass per unit leaf area as LMA_Fresh_ = 100^2^ × *M*_fresh_/*A*_leaf_, the dry matter per unit leaf area (g DM m^−2^) as LMA_DM_ = 100^2^ × *M*_dry_/*A*_leaf_, and the water per unit leaf area (g H_2_O m^−2^) as LMA_H2O_ = 100^2^ × (*M*_fresh_ – *M*_dry_)/*A*_leaf_. Leaf water content (% water) was then calculated as leaf water content (LWC) = 100 × (*M*_fresh_ – *M*_dry_)/*M*_fresh_.

## Results

### Leaf morphology

**Fig. 1:**
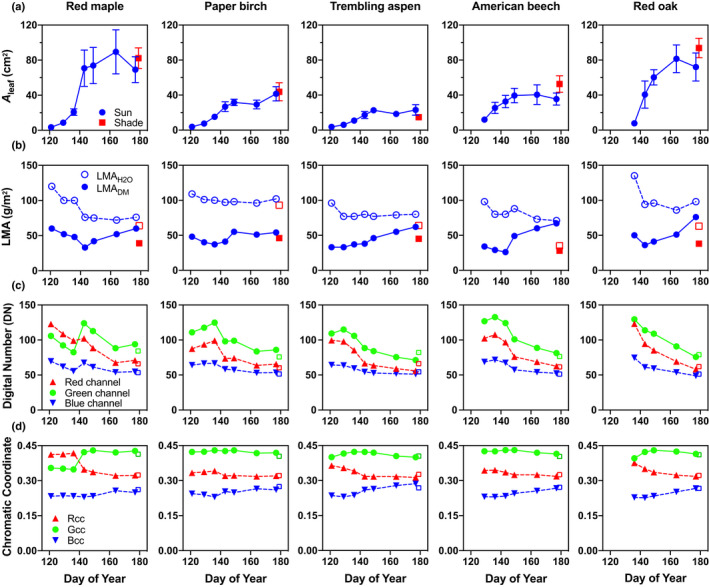
Changes in leaf morphology and composition as leaves matured. ‘Sun leaves’ were collected from outer canopy, high light environments over an 8‐wk period following emergence, while ‘shade leaves’ were collected from shaded microenvironments (one collection only). Error bars indicate ± 1 standard deviation across *n* = 3 replicate leaves for each collection date and canopy position. (a) *A*_leaf_ is the one‐sided surface area of an individual leaf (cm^2^). (b) LMA is leaf mass per unit leaf area (g m^−2^); LMA_DM_ is the amount of dry leaf matter per unit leaf area and LMA_H2O_  is the amount of leaf water per unit leaf area; (c) Leaf colour is characterised by the intensity of the red, green and blue colour channels, as measured on a digital flatbed scanner, with intensity reported as an 8‐bit digital number for each channel (binary integer). (d) *R*_CC_, *G*_CC_ and *B*_CC_ are red, green and blue chromatic coordinates, respectively (unitless).

The most evident morphological change with leaf development was the marked expansion that occurred as leaves matured during the 8 wk of this study (Fig. 1, top row). Leaves were as small as 4 cm^2^ (per leaf) at the date of first collection, but as much as 20× larger by the date of last collection. Red maple and red oak leaves matured to an average size of 80 cm^2^, while mature trembling aspen leaves were much smaller, reaching only 20 cm^2^. As leaves expanded, the amount of dry matter per unit leaf area (LMA_DM_) also generally increased, while the amount of water per unit leaf area (LMA_H2O_) generally decreased (Fig. 1, second row). This led to a progressive drop in LWC (%) as leaves developed, particularly for American beech, in which leaf water content dropped from 75% at the beginning of the study to just over 50% by the end of the study.

Leaves also gradually changed colour as they developed. In absolute terms, the intensity of each colour band tended to decrease over time (Fig. 1, third row), and thus immature leaves were lighter in colour, and mature leaves were darker. Total intensity, as the sum of (red + green + blue) digital numbers, dropped by 45% for red oak over the course of the study. For paper birch, the corresponding drop was 25%.

We normalised each colour band against total intensity to yield a relative measure of leaf colour, independent of the darkening trend. These data show that darkening generally did not affect the relative greenness of the leaves, as the green chromatic coordinate (*G*_cc_) varied relatively little over time for most species (Fig. 1, bottom row). However, red maple was an obvious exception to this pattern. After the first 3 wk of sampling, during which time leaves were more red than green, there was a marked increase in *G*_cc_, and leaves remained more green than red for the remainder of the study period. This most likely represented a shift in the dominant leaf pigments as development occurred, from red anthocyanins to green chlorophylls.

### Leaf reflectance

**Fig. 2:**
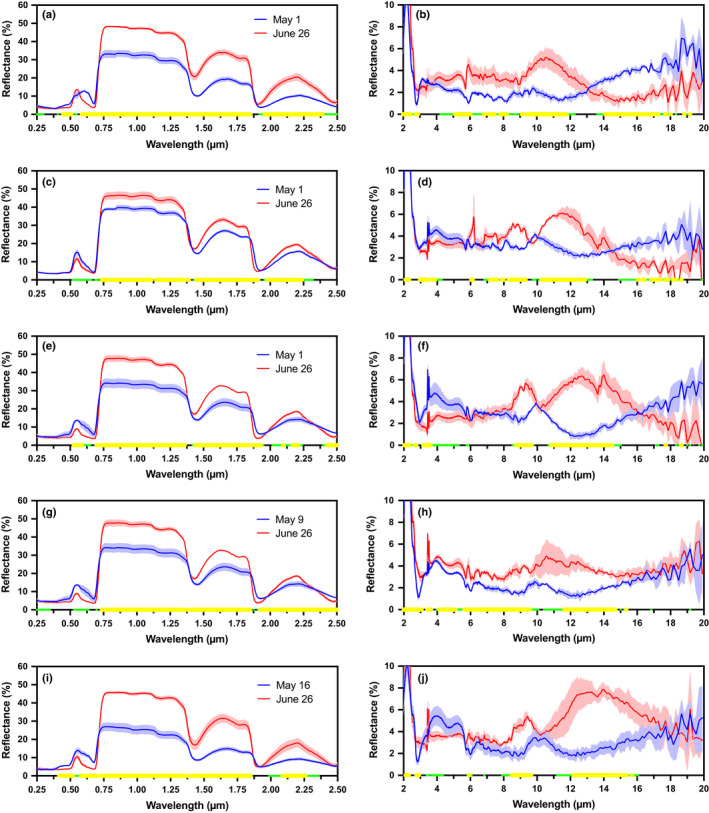
Changes in sun leaf reflectance between the first and last sample collection dates. The left column illustrates the UV‐VIS‐NIR spectra, the right column the MIR spectra. Species are: (a, b) red maple; (c, d) paper birch; (e, f) trembling aspen; (g, h) American beech; and (i, j) red oak. Solid lines and lighter shading indicate mean ± 1 standard deviation across *n* = 3 replicate sun leaf samples collected on each date. Highlighting on the *x*‐axis indicates regions of statistically significant differences between the two collection dates, with smaller green circles indicating *P* ≤ 0.05, and larger yellow circles indicating *P* ≤ 0.01, based on two‐tailed *t*‐tests, assuming equal variance.

For all five species, the UV‐VIS‐NIR reflectance spectra exhibited familiar spectral features (Fig. 2, left column), including a green peak around 550 nm, a strong red edge transition around 700 nm, a plateau from 800 to 1300 nm, and broad water absorption features at 1400, 1900 and 2600 nm. Across all species, VIS reflectance was generally lower in mature leaves (last collection, late June) than immature leaves (first collection, early May), while NIR reflectance was higher in mature leaves than immature leaves.

By comparison, the shape and level of MIR reflectance spectra tended to vary not only among species but also between immature and mature leaves (Fig. 2, right column). We note that the biological variation across replicate samples (1 SD = 0.5%) was roughly four‐fold larger than the underlying random measurement uncertainty, but that the developmental change over time was still almost an order of magnitude larger than that (1 SD ≈ 4%; described in Methods S1). Across the MIR spectrum, the variability across species means (1 SD) ranged from 0.5% to 2.5%, depending on wavelength, with generally less variability in the 5–10 µm range, and more variability at both shorter and longer wavelengths. In most species (but not red maple), there was a pair of prominent reflectance spikes at 3.4 µm and 3.5 µm; in other species these particular features have been attributed to aliphatic compounds in the cuticle (Heredia‐Guerrero *et al*., 2014). A prominent reflectance spike was observed at 6.2 µm in mature, but not immature, paper birch leaves; this may similarly be associated with phenolic compounds in the cuticle (Heredia‐Guerrero *et al*., 2014). Immature leaves commonly had a shallow reflectance peak at 10 µm, whereas in mature leaves there was a well defined reflectance trough at 10 µm in three (paper birch, trembling aspen, red oak) out of five species. The mature leaves of these three species also featured a reflectance peak at about 9 µm, which was not observed in the mature leaves of red maple or American beech. In the mature leaves of all species, there was a broad but pronounced reflectance peak within the range 10–14 µm, with reflectance in the 4–8% range, compared with 1–3% in immature leaves.

**Fig. 3:**
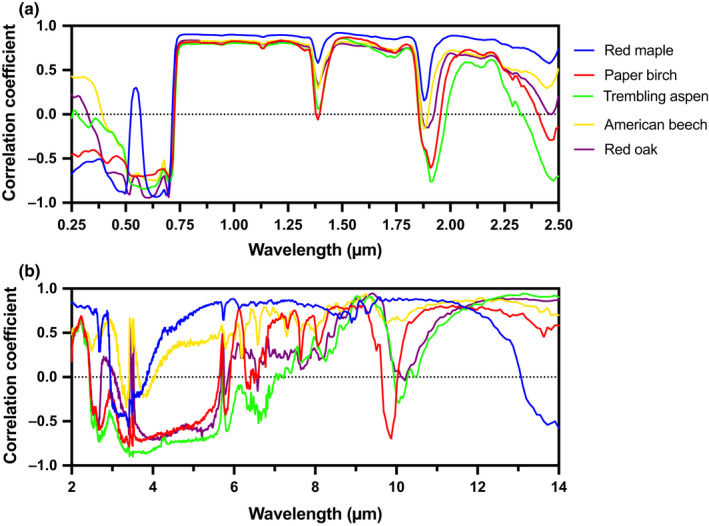
Correlation of leaf reflectance with collection date, a proxy for leaf development. A positive correlation indicates reflectance at that wavelength increased as leaf development proceeded. Panel (a) illustrates the UV‐VIS‐NIR spectrum, and (b) the MIR spectrum. The correlation coefficient is significant at *P* ≤ 0.05 for |*r*| > 0.43, and *P* ≤ 0.01 for |*r*| > 0.55 for red maple, paper birch and trembling aspen (*n* = 21 samples total, across seven sampling dates); significant at *P* ≤ 0.05 for |*r*| > 0.47, and *P* ≤ 0.01 for |*r*| > 0.59 for American beech (*n* = 18 samples total, across six sampling dates); and significant at *P* ≤ 0.05 for |*r*| > 0.52, and *P* ≤ 0.01 for |*r*| > 0.64 for red oak (*n* = 15 samples total, across five sampling dates). Only sun leaf spectra were used in this analysis.

The features and patterns described above generally correspond to statistically significant differences in reflectance between leaves from the first collection date and last collection date (two‐tailed *t*‐test at each wavelength, assuming equal variance, with *n* = 3 replicate leaf samples for each of the two sampling dates) (Fig. 2). To investigate these patterns further and better identify commonalities across species, we also conducted an analysis of the linear (Pearson's) correlation between reflectance at each wavelength and the sample collection date, using data from all sample collection dates (Fig. 3). This analysis shows that in the UV‐VIS‐NIR spectral region, reflectance from 400  to 680 nm decreased as development proceeded (*r* ≈ −0.50 to −0.90), whereas reflectance from 750 to 2500 nm increased as development proceeded (*r* ≈ +0.80 to +0.90) except for within the three water absorption features described above. Within the MIR spectral region, the patterns were more species specific. For example, from 3 to 6 µm, reflectance was negatively correlated with sample collection date (*r* ≈ −0.50 to −0.85) for three species (trembling aspen, paper birch and red oak). By comparison, from 4 to 6 µm reflectance was positively correlated with sample collection date (*r* ≈ +0.40 to +0.85) for the other two species. From 6 to 14 µm, reflectance was for the most part positively correlated with sample collection date in all species. Red maple and American beech were the best examples of this pattern; in both species the correlation of reflectance with sample collection date was consistently very strong (*r* ≥ +0.80) across most, if not all, of this spectral range. By comparison, for the other three species, there were regions of both strong (*r* ≥ +0.60 from 8.5 to 9.5 µm, and from 11 to 14 µm) and weak (*r* ≈ 0.00 around 10 µm) correlation. Overall, therefore, there was a clear relationship between leaf maturation and increasing reflectance across much of the MIR in the leaves of our five study species. The narrowness of some of the features in the correlation spectra can most likely be attributed to the increasing or decreasing abundance (in relative terms) of specific biochemical compounds at or very near the leaf surface (see Türker‐Kaya & Huck, 2017).

### Sun and shade leaves

Shade leaves differed from sun leaves in terms of morphology and colour (Fig. 1) and, while UV‐VIS‐NIR reflectance differences were negligible, in some species there were more pronounced differences in MIR reflectance. For further details, see Notes S1; Fig. S5.

### Diffuse and specular components of total MIR reflectance

Total leaf reflectance in the MIR was dominated by the diffuse component, with the specular component typically accounting for only *c*. 10% of total reflectance. For further details, see Notes S2; Fig. S6; Table S4.

### Spectral indices

**Fig. 4:**
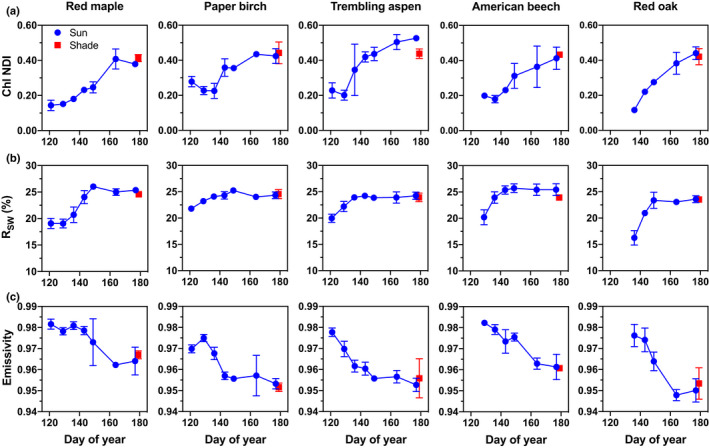
Changes in leaf reflectance indices as leaves matured. ‘Sun leaves’ were collected from outer canopy, high light environments over an 8‐wk period following emergence, while ‘shade leaves’ were collected from shaded microenvironments (one collection only). Error bars indicate ± 1 standard deviation across *n* = 3 replicate leaves for each collection date and canopy position. (a) Chl NDI (unitless) is the chlorophyll normalised difference index, calculated as (*R*_750_– *R*_705_)/(*R*_750_ + *R*_705_), where *R*_λ_ is reflectance at wavelength *λ* (in nm). (b) *R*_SW_ (%) is mean shortwave (250–2500 nm) reflectance, weighted by the solar irradiance spectrum (ASTM G173‐03) to approximate albedo. (c) Emissivity (unitless) is calculated as 1 minus the mean reflectance from 8 to 14 µm.

Chl NDI indicated virtually monotonic increases in chlorophyll content (leaf area basis) as leaves of all five species matured (Fig. 4, top). Based on calibration curves published by Richardson *et al*. (2002), we estimate that the observed increase in Chl NDI from immature (Chl NDI ≈ 0.10) to mature (Chl NDI ≈ 0.50) leaves corresponds to a seven‐fold increase in chlorophyll content, from *c*. 0.005 mg cm^−2^ to 0.035 mg cm^−2^. At the same time, total SW reflectance increased from immature (*R*_SW_ ≈ 20%) to mature (*R*_SW_ ≈ 25%) leaves. By comparison, *ε* decreased steadily as leaves of all five species matured and the mean reflectance across the range from 8 to 14 µm increased (Fig. 4, bottom). *ε* of newly expanded leaves was sometimes higher than 0.98 (e.g. red maple, American beech), while *ε* of mature leaves was in some cases lower than 0.95 (e.g. red oak).

The correlation of mean *ε* with collection date (*r* ≈ −0.91 ± 0.04; mean ± 1 SD across *n* = *5* species), LWC (*r* = 0.88 ± 0.07), Chl NDI (*r* = −0.93 ± 0.02), and SW reflectance (*r* = −0.75 ± 0.14) was generally strong in all species (Table 1). However, *ε* was not as well correlated with LMA_Fresh_, LMA_DM_, or LMA_H2O_ (Table 1).

**Table 1:** Correlation of leaf emissivity with leaf properties that changed with maturation.

Species	Collection date	LMA_Fresh_ (g m^−2^)	LMA_DM_ (g m^−2^)	LMA_H2O_ (g m^−2^)	LWC (% water)	Chl NDI	R_SW_
Red maple	−0.91	0.54	−0.10	0.77	0.93	−0.95	−0.75
Paper birch	−0.87	−0.10	−0.59	0.62	0.76	−0.92	−0.73
Trembling aspen	−0.87	−0.22	−0.79	0.68	0.92	−0.92	−0.93
American beech	−0.98	0.27	−0.49	0.79	0.90	−0.93	−0.54
Red oak	−0.91	0.27	−0.45	0.65	0.91	−0.95	−0.79
Mean ± 1 SD	−0.91 ± 0.04	0.15 ± 0.31	−0.48 ± 0.25	0.70 ± 0.07	0.88 ± 0.07	−0.93 ± 0.02	−0.75 ± 0.14

Values indicate Pearson's correlation (*r*), calculated across *n* = 24 samples (eight collections, three leaves per collection) for red maple, paper birch and trembling aspen; *n* = 21 samples (seven collections, three leaves per collection) for American beech; and *n* = 18 samples (six collections, three leaves per collection) for red oak. Here the number of collections includes multiple ‘sun leaf’ collections on different dates over an 8‐wk period, and one ‘shade leaf’ collection at the end of the study. Bottom row shows the mean correlation ± 1 standard deviation (SD), calculated across species. Emissivity was calculated across the range 8–14 µm.

LMA_Fresh_, fresh leaf mass per unit leaf area; LMA_DM_, the amount of dry leaf matter per unit leaf area; LMA_H2O_, the amount of leaf water per unit leaf area; LWC, leaf water content, as a % of fresh weight; Chl NDI, the chlorophyll normalised difference index; *R*_SW_, total shortwave reflectance.

### Reflectance of dried leaves

To investigate how LWC and leaf structure influenced the MIR spectral patterns described above, we conducted additional scans of a single mature leaf (per species) that had been: (i) oven dried overnight but otherwise intact; or (ii) oven dried and then ground to a fine powder.

**Fig. 5:**
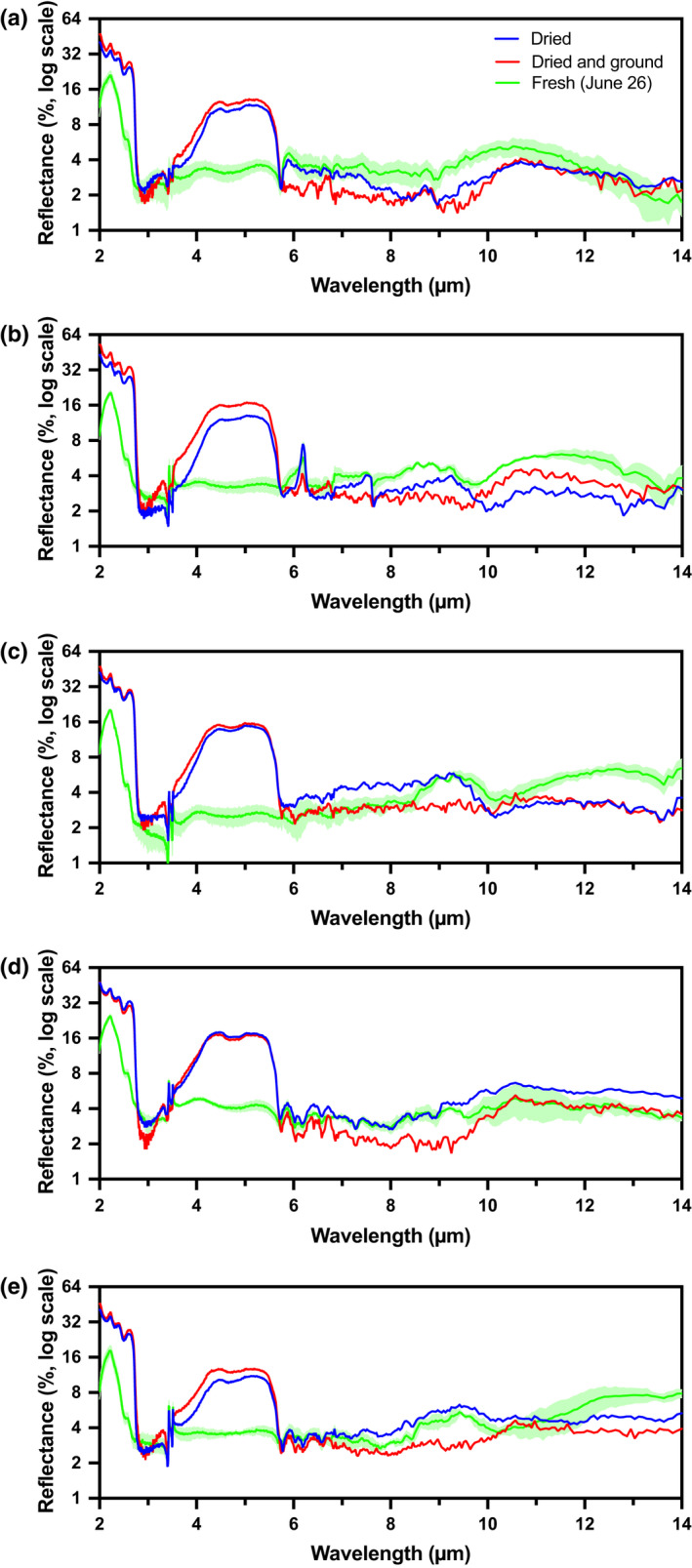
Differences in MIR reflectance spectra for fresh, dried, and dried and ground leaf samples from five temperate deciduous species. Species are as follows: (a) red maple; (b) paper birch; (c) trembling aspen; (d) American beech; and (e) red oak. For fresh leaves, the solid line and lighter shading indicates the mean ± 1 standard deviation across *n* = 3 replicate leaf samples collected on June 26. For dried, and dried and ground samples, only one scan was made per species. The *y*‐axis is on a log scale (base 2) to highlight low‐reflectance variability.

Drying resulted in marked increases in leaf reflectance from 2.0 to 2.8 µm and from 3.6 to 5.7 µm (Fig. 5). The resulting broad peaks had the highest levels of reflectance (*c*. 30–40% and *c*. 10–20%, respectively) of any spectral region from 2 to 14 µm. At wavelengths longer than 6 µm, however, there was comparatively little change in overall reflectance as a result of drying, and no obviously consistent patterns across species. Across all five species, *ε* was essentially unchanged (difference of −0.005 ± 0.011, mean ± 1 SD across species; difference not significant at *P* = 0.31, by two‐tailed paired *t*‐test) between fresh (0.956 ± 0.006) and dried leaves (0.962 ± 0.011) (Table 2).

**Table 2:** Difference in emissivity between fresh and dried leaf samples

Species	Fresh leaf emissivity	Dried leaf emissivity	∆Emissivity (fresh – dried)
Red maple	0.964 ± 0.007	0.973	−0.009
Paper birch	0.953 ± 0.002	0.971	−0.018
Trembling aspen	0.953 ± 0.003	0.962	−0.009
American beech	0.961 ± 0.006	0.951	0.010
Red oak	0.950 ± 0.006	0.951	−0.001
Mean ± 1 SD	0.956 ± 0.006	0.962 ± 0.011	−0.005 ± 0.011

For fresh leaves, reported value is mean ± 1 standard deviation based on *n* = 3 leaf samples. For dried leaves, only one sample per species was scanned. Bottom row shows mean emissivity ± 1 standard deviation (SD), calculated across species. Emissivity was calculated across the range 8–14 µm.

By comparison, the MIR spectra of dried and ground leaves tended to be reasonably similar across species (Fig. 5). The broad peaks from 2.0 to 2.8 µm and from 3.6 to 5.7 µm that had been associated with drying were observed in the samples that were also ground. In all species, reflectance of ground samples was generally low from 6 to 10 µm, with a broad, but somewhat inconspicuous, reflectance peak from 10 to 12 µm. The pair of prominent reflectance spikes at 3.4 and 3.5 µm, which had been observed in reflectance spectra of mature fresh leaves for four of five species, was much less apparent in dried and ground leaf samples.

## Discussion

We conducted an extensive set of measurements of leaf reflectance across the spectrum from ultraviolet to thermal bands (250 nm to 20 µm). Our results indicated marked differences in leaf reflectance both among species and as leaves of all species developed (Fig. 2). The most interesting spectral differences were observed to occur within the MIR. For most leaves the UV‐VIS‐NIR reflectance spectrum was generally similar, with some variation in magnitude but little variation in shape. By comparison, we found that within the MIR there was substantial variation in both magnitude and shape, across species and over time. But, for leaves collected on the final sampling date, the beech and red oak MIR spectra we measured were extremely similar to those published for the same species in previous studies (e.g. Salisbury & Milton, 1988). Patterns of variation in MIR reflectance can be explained by variation across species and over time in cuticular composition and thickness (e.g. Heredia‐Guerrero *et al*., 2014), as we explore in the following section.

### Reflectance differences over time and across species

To assist interpretation of the MIR reflectance spectra and the associated sources of variation, we re‐plotted the 6–14 µm data from Figs 2 and 7 in Fig. S7, with spectra grouped according to the nature of the leaf material that was scanned. The spectra of immature fresh leaves were surprisingly similar across species (Fig. S7a) and, in fact, generally similar to the typical ligno‐cellulose spectrum described by Elvidge (1988). This can be attributed to the thin, relatively undeveloped cuticle of newly unfolded leaves. As a result, the structural constituents of the underlying epidermal cell wall – predominantly cellulose, hemicellulose, lignin and pectin – contributed substantially to the reflectance spectra. By comparison, the spectra of mature fresh leaves were very different across species (Fig. S7b) with the ligno‐cellulose signature tending to disappear as the cuticle developed and thickened. Additionally, narrow reflectance features known to be associated with cuticular compounds became more defined as foliage matured. These included features at 3.4 µm and 3.5 µm (Fig. 2), which have been associated with aliphatic material in the cuticle, for example cutin, waxes and cutan (Heredia‐Guerrero *et al*., 2014). The fact that changes in MIR reflectance over time tended to be variable among species (Fig. 3) is interpreted as indicative of the variation among species in patterns of cuticular development (Jeffree, 2007), and the associated diversity in biochemical composition and structure of the mature cuticle among species (Salisbury, 1986; Holloway, 1994; Ribeiro da Luz, 2006). Developmental changes in MIR reflectance translated to a decrease in *ε* as foliage matured, and we found that variation in *ε* correlated with variation in a number of other leaf properties (Table 1). However, given the shallowness of MIR penetration into the leaf, these correlations are not presumed to indicate causal relationships, but rather concurrent developmental changes.

There have been comparatively few other measurements of MIR reflectance over the course of leaf development. Gates & Tantraporn (1952) reported that 7.5, 10, and 15 µm reflectance of a mature *Ulmus* leaf was almost double that of an immature elm leaf. By comparison, Wong & Blevin (1967) could not detect substantial differences in MIR reflectance between juvenile and mature leaves of several species. While Ribeiro da Luz (2006) identified MIR reflectance differences (using the method of attenuated total reflectance (ATR)) over the growing season from May to September, the spectral changes observed as leaves aged were smaller than reported here. By focusing here on the first 8 wk of leaf development, during which time development of the cuticular membrane is largely completed (Hull *et al*., 1975), we have been able to identify marked differences across species in the nature of developmental changes in MIR reflectance.

Finally, we found that MIR reflectance differences among species persisted with oven drying (Fig. S7c), although some new spectral features at wavelengths less than 6 µm were also observed in dried leaves (Fig. 5). Salisbury & Milton (1988) noted that drying leaves had little effect on reflectance from 8 to 14 µm. In our analysis, the observed differences between fresh and dried leaf spectra at these longer wavelengths may have resulted from softening and reorganisation of the cuticular and epicuticular waxes in the 60°C oven. However, differences among species essentially disappeared with leaf grinding (Fig. S7d), so that the spectra of dried and ground leaves tended to again converge on Elvidge’s (1988) ligno‐cellulose spectrum. Grinding destroys leaf and cell structure and should permit the main leaf structural constituents – which are essentially shielded by the cuticle in intact, unground leaf samples – to dominate MIR reflectance. The general similarity of MIR reflectance from the dried and ground samples can thus be attributed to the similar chemical make‐up of the cell wall across species (similar to our proposition for newly expanded fresh leaves, which exhibited a roughly comparable spectral signature; Fig. S7a).

### Variation in leaf emissivity

Over the 2 months following leaf emergence, leaf *ε* of the five species studied here exhibited a steady decline as leaves matured, from a maximum *ε* of almost 0.99 to a minimum *ε* of about 0.95. Once leaves had matured, differences across species in *ε* were relatively small (Table 2). Because of the comparatively limited ecological variation among our five co‐occurring study species, this lack of variability may not be surprising, but it does raise questions about what sort of variation might be expected in other ecosystem types or climate regimes. But, it is likely to be not possible to predict, based on reflectance in other spectral regions, what MIR reflectance of a given species might look like because the physical properties driving leaf reflectance differ among spectral regions.

Of the handful of papers that present MIR reflectance or *ε* measurements, most have focused on temperate species. There has been extremely limited sampling of species that are not broadleaf temperate woody plants. Intriguingly, however, the data from more diverse taxa do not indicate substantially more variation than we found here in relation to leaf development. Crops in Spain (López *et al*., 2012) and India (Pandya *et al*., 2013) have been shown to have leaf *ε* that ranges from 0.95 to 0.98. Arp & Phinney (1980) measured branch‐level *ε* of a wide variety of plants in a wide range of ecosystem types (from cloud forest to desert) in the USA and Mexico, and reported that *ε* of desert plants (0.981 ± 0.011, mean ± 1 SD) was somewhat higher than that of rainforest (0.962 ± 0.020) or temperate (0.977 ± 0.012) plants. Thus we conclude that the change in *ε* that occurred as foliage matured may be almost as large as the variation that has been previously observed across different plant functional types and climate regimes.

### Implications for leaf temperature

Leaf development is therefore associated with changes in leaf reflectance that have the potential to influence leaf energy balance and hence leaf temperature. Total shortwave reflectance was higher, and thermal emissivity lower, in mature leaves compared with immature leaves (Fig. 4). Thus, as leaves mature they become less like blackbody emitters. First‐order insight into the effects of changes in leaf *ε* on leaf temperature can be obtained by considering just the longwave components of the equation for net radiation, specifically the balance between the longwave emitted by the leaf ($2\varepsilon \sigma T_{\text{leaf}}^{4}$) and the longwave incident upon and absorbed by the leaf $\left(\alpha \sigma \left(T_{\text{sky}}^{4}+T_{\text{surf}}^{4}\right)\right)$. Here, *α* is the longwave absorptance of the leaf (equal to *ε*), *σ* is the Stefan–Boltzmann constant (5.67 × 10^−8)^), and *T* denotes the relevant temperature (in K). This representation assumes a horizontal leaf, emitting longwave from both its upward‐ and downward‐facing sides, with the bottom side exposed to a land surface of temperature *T*_surf_ and the upper side of the leaf exposed to a sky of temperature *T*_sky_.

As a simple example, assume that the land surface temperature equals the air temperature (25°C or *c*. 298 K), the sky is 20 K below air temperature (*c*. 278 K), and the leaf is 5 K above air temperature (*c*. 303 K). Then, for *ε* = 1, the longwave incident upon the leaf from the sky is 339.4 W m^−2^ and from the surface is 448.0 W m^−2^, while 478.9 W m^−2^ is emitted by each side of the leaf. The net flux is thus −170.3 W m^−2^, representing a net flow of longwave from the leaf to its surroundings. By comparison, for *ε* = 0.99, the same calculations yield a net flux of −168.6 W m^−2^. These results indicate a difference of −1.7 W m^−2^ per +0.01 change in *ε*, and hence a cooler leaf under equilibrium conditions for higher *ε*. Relatively similar results are obtained with other reasonable assumptions about leaf and surface temperatures relative to air temperature. The effect of *ε* on the longwave energy balance is thus small. It is also linear.

To estimate the equilibrium effects of changes in *ε* on *T*_leaf_ we used the tealeaves v.1.0.1 R package (Muir, 2019). This analysis showed that a 0.01‐unit increase in longwave *ε* leads to a *c*. 0.055 K decrease in leaf temperature under typical, midday summertime conditions. By comparison, a 0.01‐unit decrease in shortwave absorptance causes a *c*. 0.25 K decrease in leaf temperature. Thus, the impact of changing shortwave absorptance has about a five‐fold greater impact on leaf temperature than the same change in *ε*. The *c*. 0.04‐unit decrease in longwave *ε* that we observed with leaf development is expected to cause only a 0.22 K increase in leaf temperature. Although we did not measure transmittance, and hence cannot quantify the effect of developmental changes in shortwave absorptance, the potential impact on leaf temperature of the *c*. 0.05% increase in shortwave reflectance that we observed here is obviously considerably larger. We conclude that from the point of view of leaf temperature, the impact of developmental changes in emissivity documented here are negligible. However, the magnitude of developmental changes in *ε* have a much larger potential impact on leaf temperature measured using radiometric measurements, as shown in the following sections.

### Implications for radiometric temperature

The developmental changes in *ε* shown here are sufficient to cause an almost 3 K error in leaf temperature measured using radiometric approaches, if *ε* is assumed to be constant, with the temperature underestimated if *ε* is overestimated, and vice versa. While the potential for these errors has been reported previously (Fuchs & Tanner, 1966; Arp & Phinney, 1980; Van de Griend *et al*., 1991), we were not aware of realistic boundaries having been placed on the likely magnitude of *ε* errors, especially in relation to leaf development effects; many studies have simply assumed that *ε* = 0.95 or 1.0. The physiological relevance of a 3 K error in leaf temperature is highlighted with two examples. First, if the air temperature is 25°C (*c*. 298 K) and relative humidity is 50% (vapour pressure = 1.6 kPa), the leaf‐to‐atmosphere VPD for a leaf at 30°C (*c*. 303 K) is 2.6 kPa vs 1.96 kPa for a leaf at 27°C (*c*. 300 K) or 3.4 kPa for a leaf at 33°C (*c*. 306 K). In other words, a difference of 3 K in leaf temperature would be associated with a difference of about 30% in estimated VPD. In this example, underestimating leaf temperature because *ε* is overestimated thus results in substantial underestimation of the evaporative demand, which would lead to incorrect estimates of transpiration. As a second example, if we assume dark respiration scales as an exponential function of leaf temperature with a Q_10_ of 2.0, then a 3 K error in leaf temperature would cause a 23% error in modelled respiration. These examples show the importance of accurate emissivity values for leaf‐level studies related to water and carbon.

Developmental effects on *ε* are most important when the scale of measurement permits resolution of individual leaves. Fuchs & Tanner (1966) recognised that the *ε* of an individual leaf was not necessarily the same as that of the canopy: canopies can have higher *ε* than the individual leaves of which they are comprised as canopy geometry can lead to a high amount of scattering and internal reflection. Because reflectance peaks are emittance troughs, this scattering results in ‘filling in’ of the troughs (Salisbury, 1986), reducing spectral contrast and causing plant canopies to function more like blackbody cavities, with canopy‐level *ε* potentially approaching unity (Van de Griend *et al*., 1991; Salisbury *et al*., 1994a). These cavity effects increased with leaf area index (Jin & Liang, 2006). Cavity effects would become problematic with coarse scale remote sensing (e.g. satellite), in which individual canopy elements cannot be distinguished. However, cavity effects are thought to be minimal for planophile canopies, particularly when the specular component is dominant (Salisbury, 1986). In general, the species studied here have been shown to have planophile canopies (Pisek *et al*., 2013), although our data showed that most MIR reflectance was diffuse rather than specular. Regardless of canopy geometry, the issue of radiometric temperature bias from inaccurate emissivity values will be an issue when individual leaves or branches are the target of measurement, for example in fine‐resolution near‐surface remote sensing applications. As has been noted by Ribeiro da Luz & Crowley (2010), the MIR spectral features of many deciduous broadleaf tree species at the canopy scale remained sufficiently unique – in spite of cavity effects – to permit species identification using hyperspectral thermal imagery. Thus it is to be expected that variation in *ε*, both over time and across species, remains ecophysiologically significant at the canopy scale.

### Conclusions

Our results have shown that from 250 to 2500 nm, spectral features are highly similar across species, whereas beyond 2500 nm there are unique features that are associated with each species. The novel contribution of this study is the finding that this interpretation is complicated by the fact that within the MIR, the reflectance spectra are dynamic in time over the course of development. Notably, young, newly expanded leaves tend to converge on the ligno‐cellulose spectra described by Elvidge (1988). Differences among species in the MIR reflectance of mature leaves are attributed (e.g. Salisbury, 1986; Ribeiro da Luz, 2006) to differences in the composition and structure of the outer layers of the leaf surface, which are magnified over time as the cuticle and epicuticular wax layers develop in thickness and complexity. Finally, our study showed that key spectral properties – chlorophyll absorption, total shortwave reflectance, and *ε* – all changed with leaf development. The developmental changes we measured in *ε* were surprisingly large – as large as have been measured across plant functional types – but would appear to be too small to have had a substantial effect on the actual leaf temperature. However, these changes are large enough to cause substantial biases (up to 3 K) in radiometrically measured leaf temperature, if variation in *ε* is not accounted for.

To conclude, our measurements and analyses showed that there is still a great deal to be learned about leaf development and function from a better understanding of the leaf spectral properties. There is also the potential to more directly link the observed variation in MIR reflectance to variation in the composition and morphology of the cuticular membrane, and thus better understand species‐level differences in cuticular development. The variation across species, within species and over time of MIR reflectance spectra are not only a scientific curiosity but also of ecophysiological relevance because of the impact of changes in emissivity on radiometric temperature at the leaf level.

## Author contributions

ADR and DMA designed the study. ADR collected samples. ADR, DMA and LH conducted measurements. ADR, DB, KH and CDM analysed data. ADR drafted the manuscript. All authors provided feedback on the manuscript.

## Supporting information


**Fig. S1** Baseline FT‐IR measurements used to calculate corrected sample reflectance and for quality assurance.
**Fig. S2** MIR (2–14 µm) reflectance spectra of high‐ and low‐reflectance reference materials.
**Fig. S3** Comparison of leaf reflectance measured by two different instruments with overlapping spectral ranges.
**Fig. S4** Reflectance measured from the external spectral exclusion cavity of the IntegratIR sphere.
**Fig. S5** Differences in leaf reflectance between sun and shade leaves of five temperate deciduous species.
**Fig. S6** Diffuse and specular reflectance spectra (2–14 µm), for sun and shade leaves of five temperate deciduous species.
**Fig. S7** MIR reflectance spectra (6–14 µm) for leaves of five temperate deciduous species.
**Methods S1** FT‐IR reflectance calculations, assumptions, baseline measurements and uncertainty estimates.
**Methods S2** Determination of diffuse and specular reflectance components.
**Notes S1** Differences between sun and shade leaves.
**Notes S2** Diffuse and specular components of total reflectance.
**Table S1** List of symbols used.
**Table S2** Uncertainty quantification.
**Table S3** Sources of variability (biological variability and developmental change) in measured leaf sample spectra.
**Table S4** Mean total reflectance (2–14 µm), partitioned to diffuse and specular components, for mature sun and shade leaves of five temperate deciduous species.
Please note: Wiley Blackwell are not responsible for the content or functionality of any Supporting Information supplied by the authors. Any queries (other than missing material) should be directed to the *New Phytologist* Central Office.
