# Supplementary material for: Developmental changes in the reflectance spectra of temperate deciduous tree leaves and implications for thermal emissivity and leaf temperature
Source: New Phytol. 2020 Nov 29;229(2):791–804. doi: 10.1111/nph.16909 (PMC7839683; doi:10.1111/nph.16909)
Supplement: Supplementary file 1 — Fig. S1 Baseline FT‐IR measurements used to calculate corrected sample reflectance and for quality assurance. Fig. S2 MIR (2–14 µm) reflectance spectra of high‐ and low‐reflectance reference materials. Fig. S3 Comparison of leaf reflectance measured by two different instruments with overlapping spectral ranges. Fig. S4 Reflectance measured from the external spectral exclusion cavity of the IntegratIR sphere. Fig. S5 Differences in leaf reflectance between sun and shade leaves of five temperate deciduous species. Fig. S6 Diffuse and specular reflectance spectra (2–14 µm), for sun and shade leaves of five temperate deciduous species. Fig. S7 MIR reflectance spectra (6–14 µm) for leaves of five temperate deciduous species. Methods S1 FT‐IR reflectance calculations, assumptions, baseline measurements and uncertainty estimates. Methods S2 Determination of diffuse and specular reflectance components. Notes S1 Differences between sun and shade leaves. Notes S2 Diffuse and specular components of total reflectance. Table S1 List of symbols used. Table S2 Uncertainty quantification. Table S3 Sources of variability (biological variability and developmental change) in measured leaf sample spectra. Table S4 Mean total reflectance (2–14 µm), partitioned to diffuse and specular components, for mature sun and shade leaves of five temperate deciduous species. Please note: Wiley Blackwell are not responsible for the content or functionality of any Supporting Information supplied by the authors. Any queries (other than missing material) should be directed to the New Phytologist Central Office. [file NPH-229-791-s001.pdf]

New Phytologist

Article title: **Developmental changes in the reflectance spectra of temperate deciduous tree leaves, and implications for thermal emissivity and leaf temperature**

Authors: Andrew D. Richardson, Donald M. Aubrecht, David Basler, Koen Hufkens, Christopher D. Muir, and Leonard Hanssen

Article acceptance date: 21 August 2020

The following Supporting Information is available for this article:

Fig. S1 Baseline FTIR measurements used to calculate corrected sample reflectance and for quality assurance.

Fig. S2 MIR (2 µm to 14 µm) reflectance spectra of high- and low-reflectance reference materials.

Fig. S3 Comparison of leaf reflectance measured by two different instruments with overlapping spectral ranges.

Fig. S4 Reflectance measured from the external spectral exclusion cavity of the IntegratIR sphere.

Fig. S5 Differences in leaf reflectance between sun and shade leaves of five temperate deciduous species.

Fig. S6 Diffuse and specular reflectance spectra (2 µm to 14 µm), for sun and shade leaves of five temperate deciduous species.

Fig. S7 MIR reflectance spectra (6 µm to 14 µm) for leaves of five temperate deciduous species.

Table S1 List of symbols used.

Table S2 Uncertainty quantification.

Table S3 Sources of variability (biological variability and developmental change) in measured leaf sample spectra.

Table S4 Mean total reflectance (2 µm to 14 µm), partitioned to diffuse and specular components, for mature sun and shade leaves of five temperate deciduous species.

Methods S1 FTIR reflectance calculations, assumptions, baseline measurements, and uncertainty estimates.

Methods S2 Determination of diffuse and specular reflectance components.

Notes S1 Differences between sun and shade leaves.

Notes S2 Diffuse and specular components of total reflectance.

Fig. S1 Baseline FTIR measurements used to calculate corrected sample reflectance and for quality assurance. These measurements were repeated at the beginning of each measurement session. Red lines indicate the mean, and grey shading the variability (95% confidence interval) across multiple measurements (*n* is the number of independent spectra measured, i.e., on different days). Note that the *y*-axis range is 15 % in panels (a) and (b), but only 5 % in panels (c) and (d). (a) reflectance spectra of a roughened gold reference standard (Pike Technologies, Madison, WI, USA). The NIST (National Institute of Standards and Technology) measurement (ρ_{st}) is taken as the "true" reflectance of the reference standard, while the NAU (Northern Arizona University) measurement (R_{st}) is the reference standard reflectance measured on the Nicolet iS10 FTIR spectrometer used in the present study; (b) reflectance spectra of a second roughened gold reference standard (Middleton Spectral Vision, Middleton, WI, USA); (c) reflectance spectra measured with no sample on the open port of the integrating sphere (R_0), to quantify port overfilling; (d) reflectance spectra measured with aluminum foil blocking the illumination beam before it enters the sphere and detector assembly. Note that for all measurements on the NAU FTIR, the comparison method was used and reference measurements were made off the wall of the instrument's gold integrating sphere. See Table S1 for a full list of symbols used.

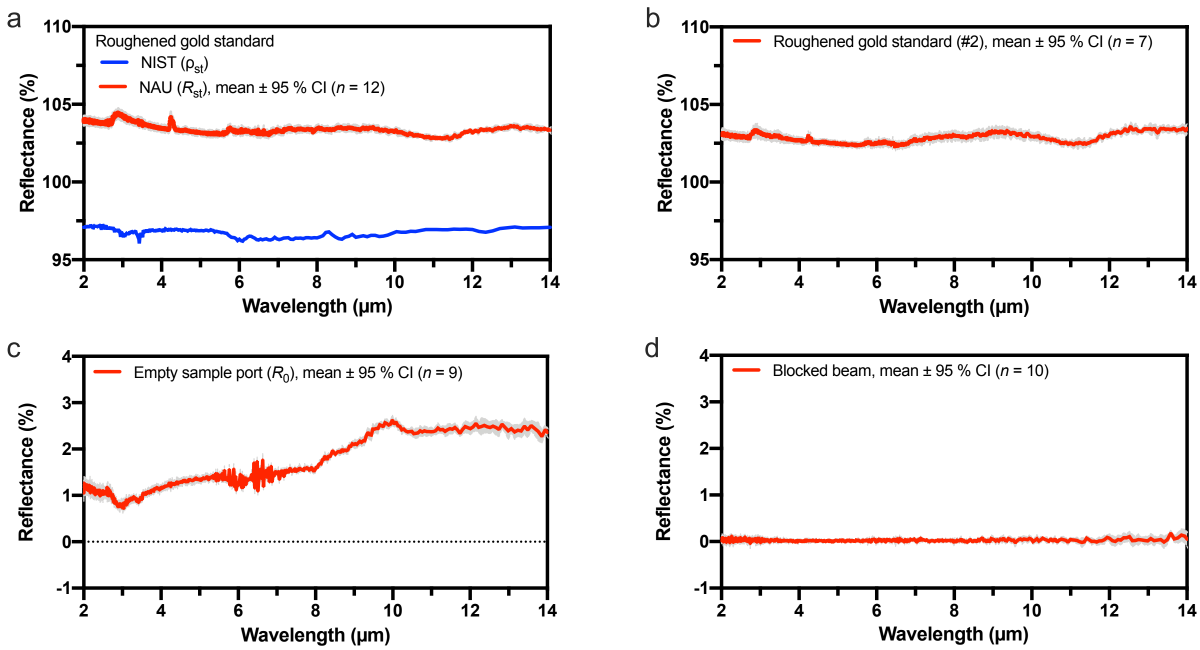

Fig. S2 MIR (2 µm to 14 µm) reflectance spectra of high- and low-reflectance reference materials. Plots show a comparison of corrected reflectance spectra (ρ_s) measured on the NAU (Northern Arizona University) Nicolet iS10 FTIR spectrometer, and as measured by NIST (National Institute of Standards and Technology). The *y*-axis range (10 %) is identical in all panels. (a) NIST roughened gold reference standard; (b) Aeroglaze Z306; (c) D25C16; (d) ESLI Velvet. Note that for all measurements on the NAU FTIR, the comparison method was used and reference measurements were made off the wall of the instrument's gold integrating sphere. Gray shading indicates 95 % confidence interval around the mean, based on n = 7 independent scans (conducted on different measurement days).

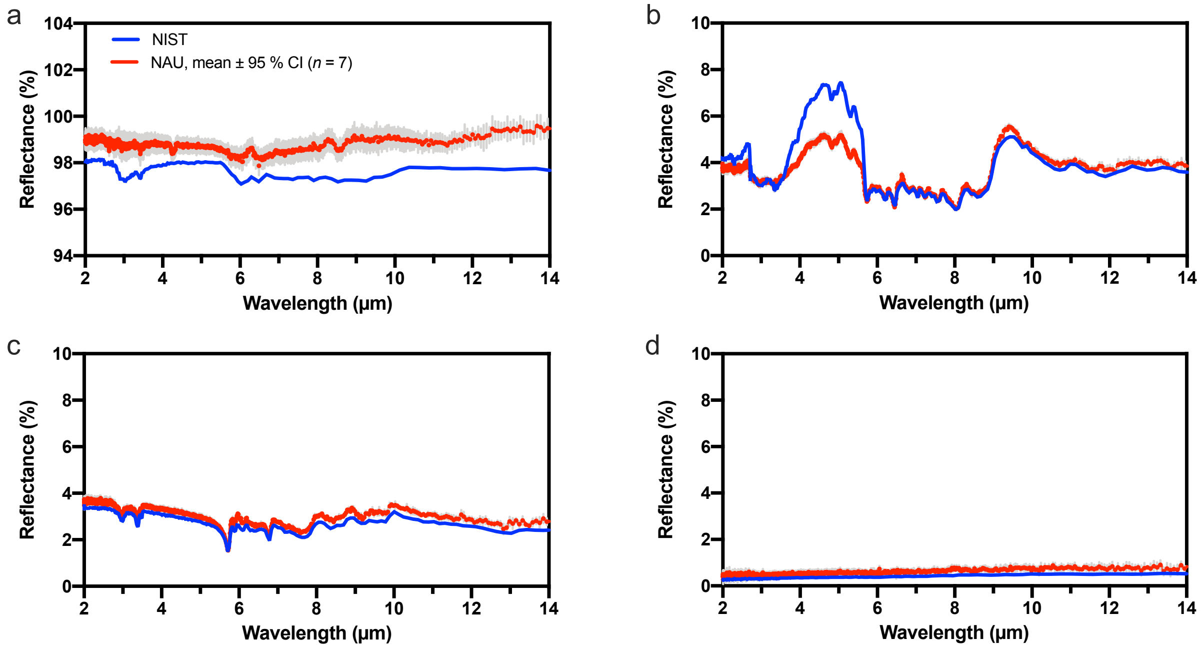

Fig. S3 Comparison of leaf reflectance measured by two different instruments with overlapping spectral ranges. The PerkinElmer Lambda 750s has a range from 0.25 µm to 2.5 µm, while the Nicolet iS10 has a range from 2.0 µm to 20 µm. (a) agreement within the overlap region between the two instruments, for lower-reflectance immature red maple leaves (collected May 1) and higher-reflectance mature red maple leaves (collected June 26); and (b) reflectance at the 2.2 µm reflectance peak, for leaves of 5 deciduous species and multiple collection dates, as measured on the Nicolet (*x*-axis) vs. PerkinElmer (*y*-axis) spectrometers. Shading around the Nicolet line in (a) and error bars in (b) indicate ± 1 standard deviation across *n* = 3 replicate leaf samples collected for each species-date combination. In (b), *n* is the number of independent species-date combinations, and *r* is Pearson's correlation.

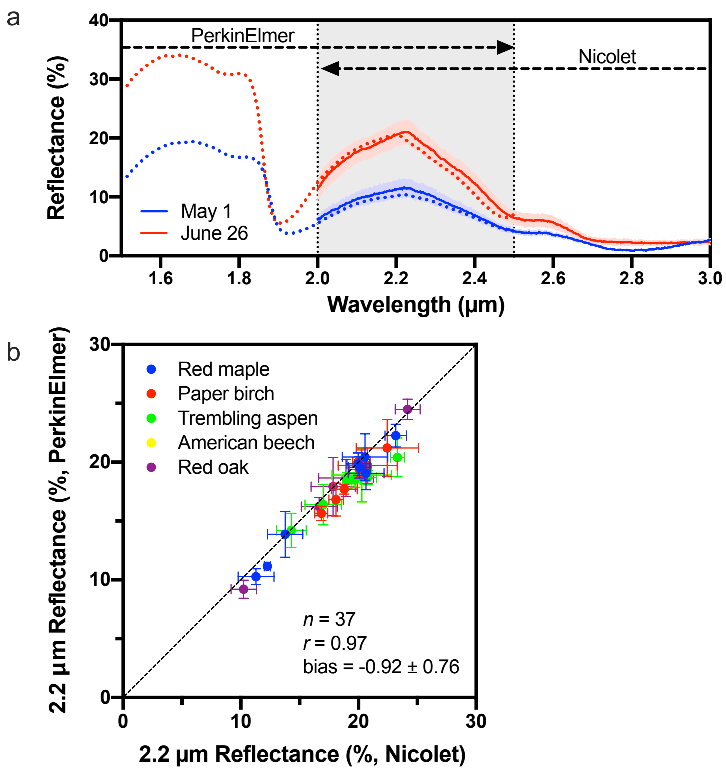

Fig. S4 Reflectance measured from the external spectral exclusion cavity of the IntegratIR sphere. Measurements were conducted with a first surface mirror on the open sample port and the spectral exclusion port in the open position. The standard coating on the external cavity is candle soot, but this material exhibited pronounced reflectance features from 2 µm to 8 µm. We filled the external cavity with a piece of ESLI Velvet and measured much lower reflectance across the entire spectrum from 2 µm to 14 µm.

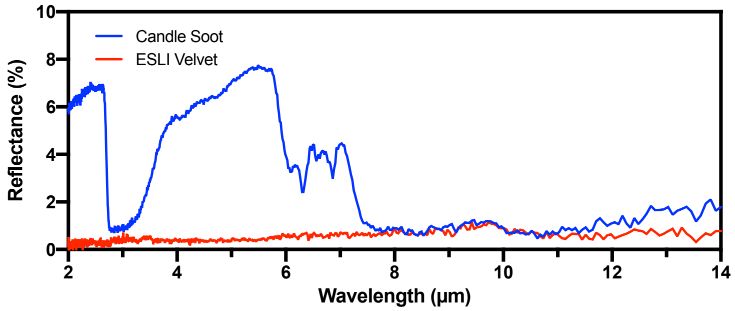

Fig. S5 Differences in leaf reflectance between sun and shade leaves of five temperate deciduous species. Species are as follows: (a, b) red maple; (c, d) paper birch; (e, f) trembling aspen; (g, h) American beech; and (i, j) red oak. UV-VIS-NIR spectra are shown in the left column (a, c, e, g, and i), and MIR spectra are shown in the right column (b, d, f, h, and j). Solid lines and lighter shading indicate mean ± 1 standard deviation across $n = 3$ replicate leaf samples collected from each canopy position on June 26. Highlighting on the *x*-axis indicates regions of statistically significant differences between the two collection dates, with smaller green circles indicating $P \leq 0.05$, and larger yellow circles indicating $P < 0.01$, based on two tailed *t*-test, assuming equal variance.

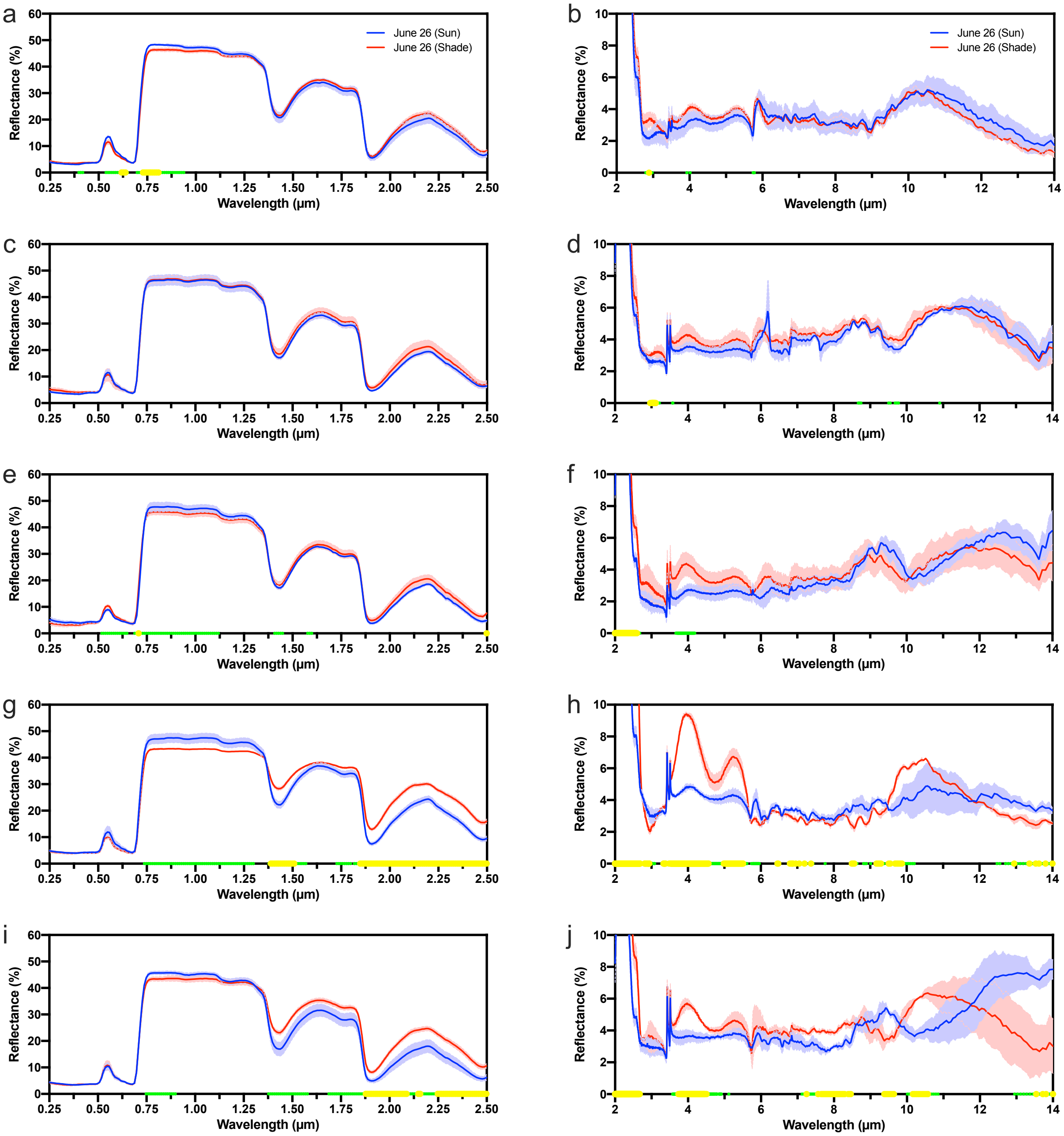

a
b
c
d
e
f
g
h
i
j
June 26 (Sun)
June 26 (Shade)
Reflectance (%)
Wavelength (µm)

Fig. S6 Diffuse and specular reflectance spectra (2 µm to 14 µm), for sun and shade leaves of five temperate deciduous species. We measured total and diffuse reflectance, and calculated specular reflectance by difference. Species are as follows: (a, b) red maple; (c, d) paper birch; (e, f) trembling aspen; (g, h) American beech; and (i, j) red oak. Sun leaf spectra are shown in the left column (a, c, e, g, and i), and shade leaf spectra are shown in the right column (b, d, f, h, and j). Solid lines and lighter shading indicate mean ± 1 standard deviation across n = 3 replicate leaf samples collected from each canopy position on June 26.

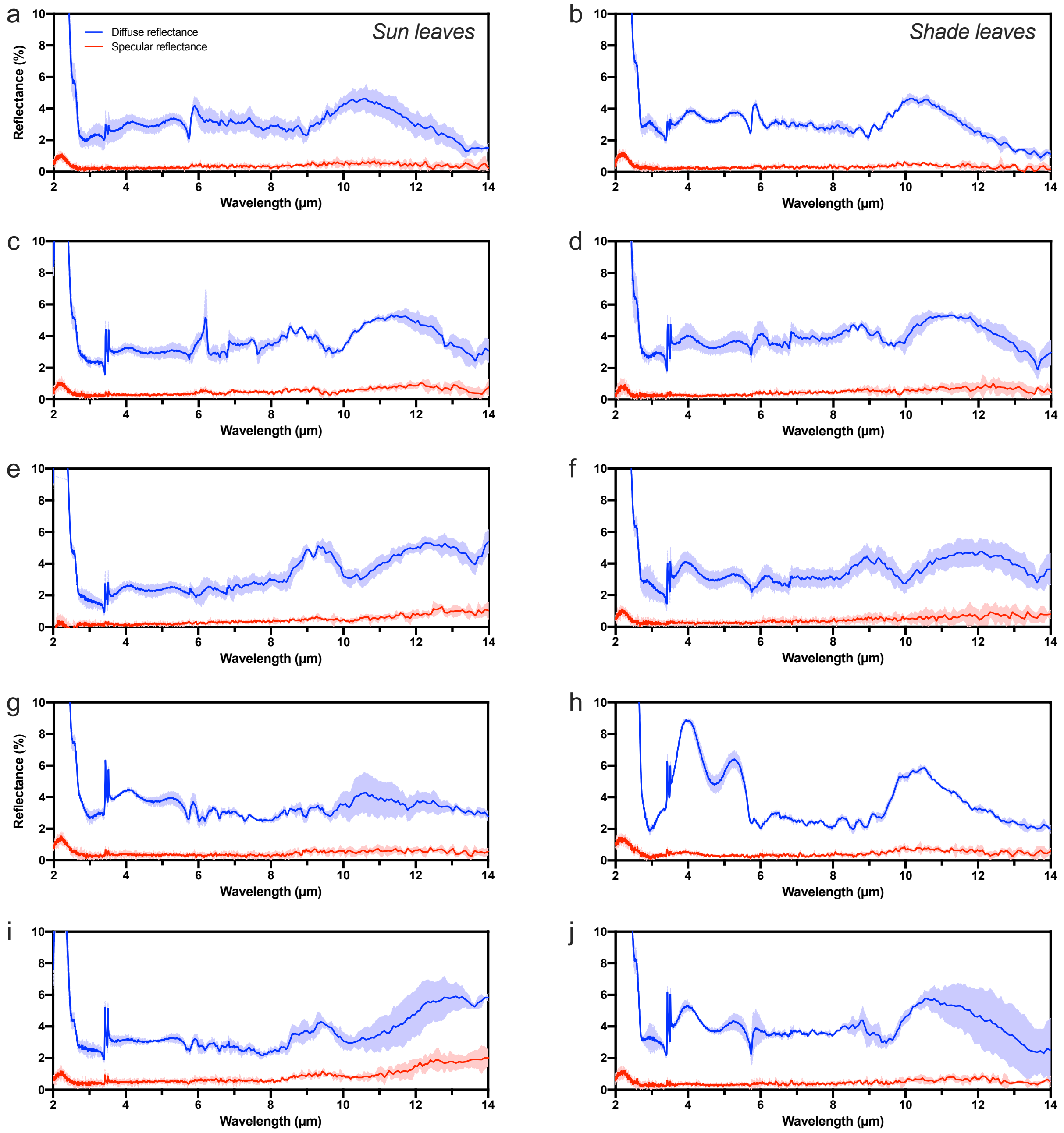

Fig. S7 MIR reflectance spectra (6 μm to 14 μm) for leaves of five temperate deciduous species. Panels are as follows: (a) immature (first collection) fresh leaves; (b) mature (last collection) fresh leaves; (c) oven-dried but intact mature leaves; (d) oven-dried and ground mature leaves. In (d), the shaded grey band shows Elvidge's (1988) ligno-cellulose spectrum, redrawn (mean ± 1 standard deviation) based on spectra presented in that paper's Figure 4 (*Arctostaphylos glauca*: gray wood, brown wood, and grey seed spectra).

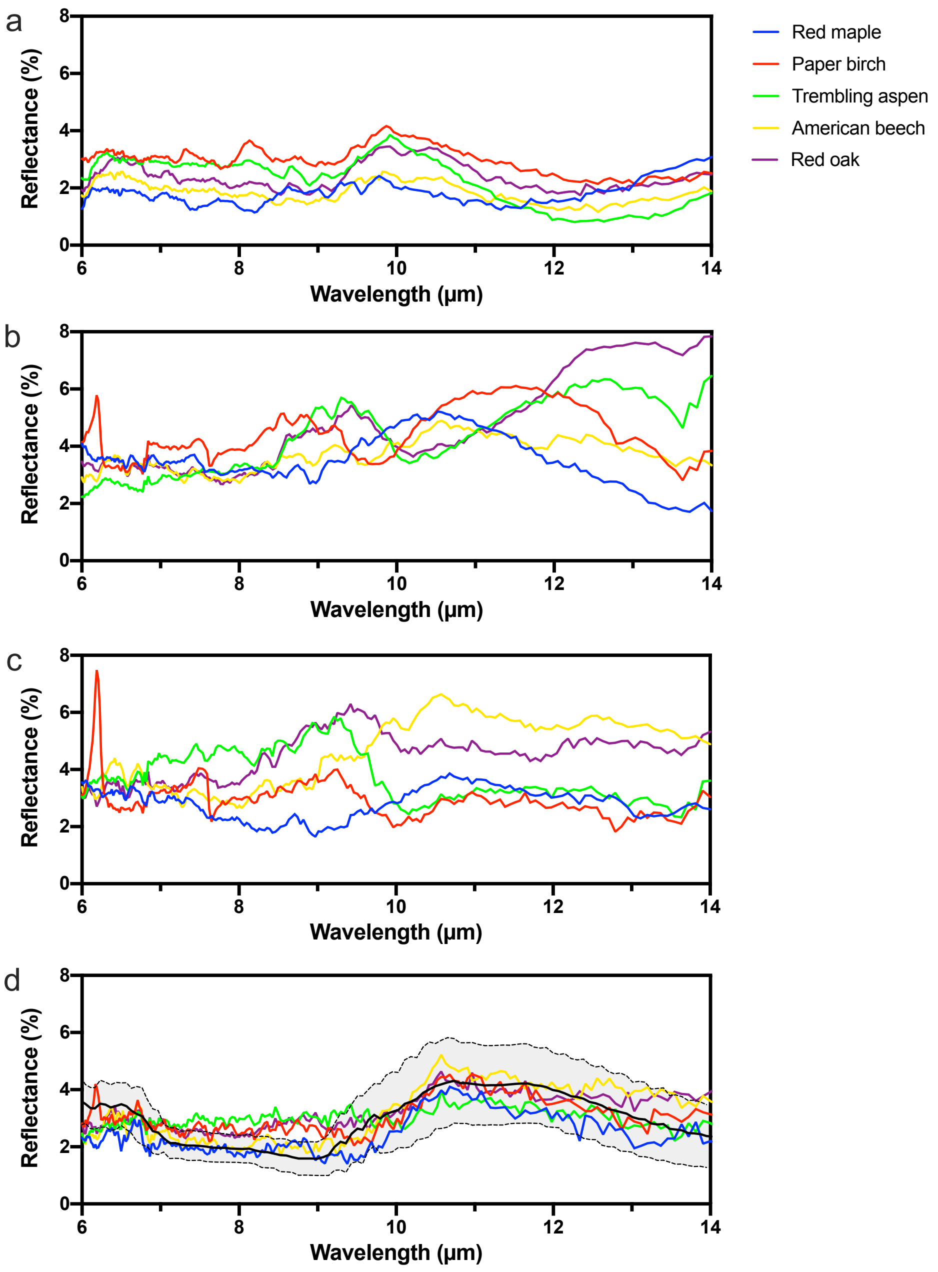

Table S1 List of symbols used.

ρ_s	sample reflectance
ρ_{ms}	directing mirror reflectance, sample measurement*
ρ_{mr}	directing mirror reflectance, reference measurement*
ρ_{st}	standard reflectance
ρ_r	reference reflectance
ρ_w	sphere wall reflectance
Φ_{in}	sphere input flux
Φ_{01s}	input flux outside of directing mirror, sample measurement*
Φ_{01r}	input flux outside of directing mirror, reference measurement*
$\rho_{ms}\Phi_s$	input flux incident on sample
$\rho_{mr}\Phi_r$	input flux incident on reference
$\rho_{ms}\Phi_{02}$	input flux within mirror, but directed outside of sample port
$\rho_{mr}\Phi_{03}$	input flux within mirror, but directed outside of reference port
τ_s	sphere throughput, from sample, sample measurement
τ_r	sphere throughput, from reference, sample measurement
τ_{ws}	sphere throughput, from sphere wall, sample measurement
τ_{st}	sphere throughput, from sample, standard measurement
τ_{rst}	sphere throughput, from reference, standard measurement
τ_{wst}	sphere throughput, from sphere wall, standard measurement
τ_{r0}	sphere throughput, from reference, zero measurement
τ_{w0}	sphere throughput, from sphere wall, zero measurement
τ_{sd}	sphere throughput for diffuse sample
τ_{ss}	sphere throughput for specular sample
c	τ_{ss} / τ_{sd}
k	detector responsivity
V_s	signal from sample measurement
V_{rs}	signal from reference measurement, with sample
V_0	signal from empty sample port measurement
V_{r0}	signal from reference measurement, with empty sample port
V_{st}	signal from standard sample measurement
V_{rst}	signal from reference measurement, with standard

* Note: the angle of incidence of input light on directing mirror is different between sample and reference measurements.

Table S2 Uncertainty quantification.

Material	NIST		NAU		
	**Mean reflectance**	**Expanded uncertainty**	**Random uncertainty**	**Systematic uncertainty**	**Expanded uncertainty**
Roughened gold standard	97.64 %	2.85 %	0.42 %	1.21 %	3.83 %
D25C16	2.70 %	0.11 %	0.12 %	0.27 %	0.60 %
ESLI velvet	0.43 %	0.02 %	0.15 %	0.21 %	0.51 %

Expanded uncertainty (coverage factor k = 2) estimates are presented for a range of standard materials that were measured by NIST (National Institute of Standards and Technology) and also on the NAU (Northern Arizona University) Nicolet iS10 FTIR spectrometer used in the present study. Random and systematic uncertainties are reported as 1 standard deviation. Reported values are means across the spectrum from 2 µm to 14 µm.

Table S3 Sources of variability (biological variability and developmental change) in measured leaf sample spectra.

Species	Biological variability	Developmental change
Red maple	0.40 %	3.12 %
Paper birch	0.42 %	4.17 %
Trembling aspen	0.44 %	4.00 %
Red oak	0.58 %	4.16 %
American beech	0.39 %	3.80 %

Assessment of biological variability (variation among replicate samples) and developmental change (variation across samples collected in the 6 to 8 weeks following bud burst) are both reported as 1 standard deviation in mean reflectance. Reported values are means across the spectrum from 2 µm to 14 µm.

Table S4 Mean total reflectance (2 μm to 14 μm), partitioned to diffuse and specular components, for mature sun and shade leaves of five temperate deciduous species.

Species	Crown position	Reflectance (%)			Specular / Total (%)
		Total	Diffuse	Specular	
Red maple	Sun	3.85 ± 0.55	3.48 ± 0.45	0.37 ± 0.10	9.5 ± 1.5
	Shade	3.85 ± 0.17	3.52 ± 0.14	0.32 ± 0.03	8.4 ± 0.4
Paper birch	Sun	4.59 ± 0.16	4.06 ± 0.08	0.54 ± 0.09	11.6 ± 1.7
	Shade	4.89 ± 0.07	4.40 ± 0.15	0.49 ± 0.14	10.0 ± 2.8
Trembling aspen	Sun	4.49 ± 0.30	4.01 ± 0.23	0.48 ± 0.12	10.7 ± 2.0
	Shade	4.62 ± 0.80	4.13 ± 0.59	0.49 ± 0.25	10.2 ± 3.6
Red oak	Sun	5.09 ± 0.37	4.13 ± 0.32	0.96 ± 0.21	18.8 ± 3.5
	Shade	5.01 ± 0.41	4.54 ± 0.47	0.47 ± 0.07	9.5 ± 2.1
American beech	Sun	4.49 ± 0.34	4.01 ± 0.23	0.48 ± 0.13	10.6 ± 2.2
	Shade	5.16 ± 0.12	4.67 ± 0.10	0.49 ± 0.04	9.4 ± 0.6

For each species and each crown position, one leaf from each of three individual trees was sampled on June 26, approximately 6 to 8 weeks after leaf-out. Reported values are mean ± 1 standard deviation, across *n* = 3 leaves.

Methods S1 FTIR reflectance calculations, assumptions, baseline measurements, and uncertainty estimates.

Calculations and assumptions

Here we describe our procedure for calculating a corrected sample reflectance measurement (ρ_s) for the Nicolet iS10 FTIR[†] scans used in this analysis. The symbols used are listed in Table S1. The corrections account for over-filling of the sample port (see also Hecker *et al.*, 2011), assessed through measurement of V_0 and V_{r0}, as well as the "true" (not equal to 100 %) reflectance of our roughened gold reflectance standard, which was quantified through independent measurement of ρ_{st} by the Optical Technology Division at the National Institute of Standards and Technology [NIST].

We begin by defining the following measured reflectances:

$R_s = V_s / V_{rs}$	measured sample reflectance
$R_0 = V_0 / V_{r0}$	measured reflectance with empty sample port.
$R_{st} = V_{st} / V_{rst}$	measured reflectance standard reflectance

The sphere input fluxes are:

$\Phi_{in} = \Phi_s + \Phi_{01} + \Phi_{02}$	sample measurement case
$\Phi_{in} = \Phi_r + \Phi_{01} + \Phi_{03}$	reference measurement case

Then the measured signals are:

$$V_s = \Phi_s \rho_{ms} \rho_s \tau_s k + \Phi_{02} \rho_{ms} \rho_w \tau_{ws} k + \Phi_{01s} \rho_w \tau_{ws} k$$
$$V_{rs} = \Phi_r \rho_{mr} \rho_r \tau_r k + \Phi_{03} \rho_{mr} \rho_w \tau_{ws} k + \Phi_{01r} \rho_w \tau_{ws} k$$
$$V_0 = \Phi_{02} \rho_{ms} \rho_w \tau_{w0} k + \Phi_{01s} \rho_w \tau_{w0} k$$
$$V_{r0} = \Phi_r \rho_{mr} \rho_r \tau_{r0} k + \Phi_{03} \rho_{mr} \rho_w \tau_{w0} k + \Phi_{01r} \rho_w \tau_{w0} k$$

Expanding $R_s = V_s / V_{rs}$:

$$R_s = \frac{\Phi_s \rho_{ms} \rho_s \tau_s k + \Phi_{02} \rho_{ms} \rho_w \tau_{ws} k + \Phi_{01s} \rho_w \tau_{ws} k}{\Phi_r \rho_{mr} \rho_r \tau_r k + \Phi_{03} \rho_{mr} \rho_w \tau_{ws} k + \Phi_{01r} \rho_w \tau_{ws} k}$$

Cancelling *k*:

$$R_s = \frac{\Phi_s \rho_{ms} \rho_s \tau_s + \Phi_{02} \rho_{ms} \rho_w \tau_{ws} + \Phi_{01s} \rho_w \tau_{ws}}{\Phi_r \rho_{mr} \rho_r \tau_r + \Phi_{03} \rho_{mr} \rho_w \tau_{ws} + \Phi_{01r} \rho_w \tau_{ws}}$$

[†] Certain commercial equipment, instruments, or materials are identified in this paper in order to specify the experimental procedure adequately. Such identification is not intended to imply recommendation or endorsement by the National Institute of Standards and Technology, nor is it intended to imply that the materials or equipment identified are necessarily the best available for the purpose.

Collecting terms:

$$R_s = \frac{\Phi_s \rho_{ms} \rho_s \, \tau_s / \tau_{ws} + (\Phi_{02} \rho_{ms} + \Phi_{01s}) \rho_w}{\Phi_r \rho_{mr} \rho_r \, \tau_r / \tau_{ws} + (\Phi_{03} \rho_{mr} + \Phi_{01r}) \rho_w}$$

Likewise for $R_0 = V_0 / \ V_{r0}$:

$$R_0 = \frac{\Phi_{02} \rho_{ms} \rho_w \tau_{w0} k + \Phi_{01s} \rho_w \tau_{w0} k}{\Phi_r \rho_{mr} \rho_r \tau_{r0} k + \Phi_{03} \rho_{mr} \rho_w \tau_{w0} k + \Phi_{01r} \rho_w \tau_{w0} k}$$

Cancelling *k* and collecting terms:

$$R_0 = \frac{(\Phi_{02} \rho_{ms} + \Phi_{01s}) \rho_w}{\Phi_r \rho_{mr} \rho_r \, \tau_{r0} / \tau_{w0} + (\Phi_{03} \rho_{mr} + \Phi_{01r}) \rho_w}$$

Applying the correction for overfilling:

$$R_s - R_0 = \frac{\Phi_s \rho_{ms} \rho_s \, \tau_s / \tau_{ws} + (\Phi_{02} \rho_{ms} + \Phi_{01s}) \rho_w}{\Phi_r \rho_{mr} \rho_r \, \tau_r / \tau_{ws} + (\Phi_{03} \rho_{mr} + \Phi_{01r}) \rho_w} - \frac{(\Phi_{02} \rho_{ms} + \Phi_{01s}) \rho_w}{\Phi_r \rho_{mr} \rho_r \, \tau_{r0} / \tau_{w0} + (\Phi_{03} \rho_{mr} + \Phi_{01r}) \rho_w}$$

Assuming the same throughput for comparison method measurements:

$$\tau_{r0} / \tau_{w0} = \tau_r / \tau_{ws}$$

Simplification yields:

$$R_s - R_0 = \frac{\Phi_s \rho_{ms} \rho_s \, \tau_s / \tau_{ws}}{\Phi_r \rho_{mr} \rho_r \, \tau_r / \tau_{ws} + (\Phi_{03} \rho_{mr} + \Phi_{01r}) \rho_w}$$

Assuming the reference region is the same as the rest of the sphere wall:

$$\rho_r = \rho_w$$

$$\tau_r = \tau_{ws}$$

Then collecting terms yields:

$$R_s - R_0 = \frac{\Phi_s \rho_{ms} \rho_s \, \tau_s / \tau_{ws}}{\big((\Phi_r + \Phi_{03}) \rho_{mr} + \Phi_{01r} \big) \rho_w}$$

Repeating for the reference measurement:

$$R_{st} - R_0 = \frac{\Phi_s \rho_{ms} \rho_{st} \, \tau_{st} / \tau_{wst}}{\big((\Phi_r + \Phi_{03}) \rho_{mr} + \Phi_{01r} \big) \rho_w}$$

Taking the ratio of the sample and reference:

$$S = \frac{R_s - R_0}{R_{st} - R_0}$$

Then:

$$S = \frac{\Phi_s \rho_{ms} \rho_s \, \tau_s / \tau_{ws}}{\left((\Phi_r + \Phi_{03})\rho_{mr} + \Phi_{01r}\right)\rho_w} \frac{\left((\Phi_r + \Phi_{03})\rho_{mr} + \Phi_{01r}\right)\rho_w}{\Phi_s \rho_{ms} \rho_{st} \, \tau_{st} / \tau_{wst}}$$

Cancelling terms yields:

$$S = \frac{\rho_s}{\rho_{st}} \frac{\tau_s}{\tau_{ws}} \frac{\tau_{wst}}{\tau_{st}}$$

If the sample and standard are both diffuse (*d*), then:

$$\frac{\tau_{s-diff}}{\tau_{ws}} = \frac{\tau_{st-diff}}{\tau_{wst}} = 1$$

Thus:

$$S = \frac{\rho_s}{\rho_{st}}$$

And:

$$\rho_{s-diff} = \rho_{st-diff} S$$

Or, if the sample and standard are both specular (*s*), then:

$$\frac{\tau_{s-spec}}{\tau_{ws}} = \frac{\tau_{st-spec}}{\tau_{wst}} = c$$

Thus again:

$$S = \frac{\rho_s}{\rho_{st}}$$

And:

$$\rho_{s-spec} = \rho_{st-spec} S$$

We note that in the present analysis, both the leaf samples and the reference standard are predominantly diffuse reflectors (Notes S2). The corrected sample reflectance ($\rho_s = S \, \rho_{st}$) is therefore calculated as:

$$\rho_s = \frac{R_s - R_0}{R_{st} - R_0} \, \rho_{st}$$

Where R_s is the measured sample reflectance, R_0 is the measured empty port reflectance, R_{st} is the measured reference standard reflectance, and ρ_{st} is the true reference standard reflectance.

Baseline measurements

At the start of measurements each day, we conducted a series of baseline FTIR measurements to enable the above calculation, and for quality assurance. We used the comparison method (Hanssen & Snail, 2001) (also referred to as the comparative method; see Hecker *et al.*, 2011), whereby the sample is placed on the open port of the integrating sphere, a reference measurement is made off the wall of the sphere using the built-in flipper mirror, and then the sample measurement is made with the flipper mirror returned to the sample position (see also Blake *et al.*, 2018). In this way, the sphere throughput is the same for both sample and reference measurements (cf. substitution method measurements, see: Hanssen & Snail, 2001; Hecker *et al.*, 2011).

The spectra from these baseline measurements are illustrated in Figure S1. We begin by comparing the measured reference standard reflectance (R_{st}) against its “true” reflectance (ρ_{st}) as measured by NIST (Figure S1a). We measured R_{st} > 100 % because the reflectance standard, a roughened gold puck, has higher reflectance than the wall of the integrating sphere. The fact that $R_{st} > \rho_{st}$ is not cause for concern; this is accounted for in the correction calculation described above. Importantly, the repeatability (on different days) of measurements was very high: expressed as a 95 % confidence interval, the variation in reflectance (grey shading around the red line) was only about 0.5 %, and consistent in magnitude across the entire spectrum from 2 μm to 14 μm. We note that a similar reflectance spectrum was also obtained for a second roughened gold reference standard which was regularly measured (Figure S1b). The empty sample port (R_0) measurement (Figure S1c) suggested a wavelength-dependent overfilling signal that varied in magnitude from ≈1 to 3 % but which was also consistently measured on different days, as the width of the 95% confidence interval was again about 0.5 %. To verify that the empty sample port measurement was not associated with other factors (e.g. stray light, electrical noise, etc.), we blocked the illuminating beam as it entered the sphere using a double layer of aluminum foil (Figure S1d). The resulting spectrum was essentially zero, with no obvious structure, across the entire range from 2 μm to 14 μm. This measurement was again highly repeatable, with very little variation across different days: the width of the 95% confidence interval was only about 0.2 %, on average across the spectrum.

Evaluation of measured reflectance for a range of reference materials

To evaluate our measurement protocol and the correction procedure described above, we compared the reflectance spectra of a variety of reference materials—both high- and low-reflectance—measured on the Nicolet iS10 FTIR spectrometer used in this study with reflectance spectra of the same samples measured by NIST. Although we measured reflectance on the FTIR from 2 μm to 20 μm, we focus here on the region from 2 μm to 14 μm, as beyond 14 μm the quality of our measurements degraded rapidly, with substantial random error (assessed by the variation across repeat, independent measurements) relative to the magnitude of the reflectance signal for low-reflectance samples.

There are several important differences to note between our setup and the NIST setup, which may help to explain the observed (but generally minor) differences between the spectra we measured and the spectra measured by NIST. The NIST setup uses a 152.4 mm (6 in) diameter integrating sphere, compared with our 76.2 mm (3 in) sphere, and the internal baffling is different. At NIST, the illumination beam is at 8° incidence, compared with 12° for our setup, which could be important if there are strong BRDF (bidirectional reflectance distribution function) effects for particular samples, or a strong specular component. At NIST, 16 to 24 repeated reflectance measurements are averaged, and the total measurement time takes up to 6 h per sample. We averaged 64 scans, but measurement of each sample was completed in under 5 min. Thus, our setup is designed to minimize per sample measurement time and prioritize convenience, at the potential expense of increased uncertainty. In the analysis below, we characterize the random and systematic errors in our measurements, and use these to develop an estimate of the expanded uncertainty.

For a roughened gold reference standard provided by NIST (Figure S2a), our corrected reflectance spectra were generally about 1 % higher (99 % reflectance vs 98 % reflectance) than the spectrum measured by NIST. Repeat measurements were somewhat noisier for this sample (95 % confidence interval width ≈1 %) compared to other high-reflectance samples (e.g. Figures S1a, b), but this noise was small relative to the overall magnitude of the reflectance (≈1 % in relative terms).

For a sample of Aeroglaze Z306 (Figure S2b), our corrected reflectance spectra were generally in very close agreement with the spectrum measured by NIST. The prominent spectral features at 2.6 µm, 4.8 µm, 8.1 µm, and 9.4 µm were seen in both spectra. But, a notable difference was that the reflectance peak from 3.6 µm to 5.7 µm was 50 % higher in the NIST spectrum (peaking at almost 7.5 % reflectance) than in our spectrum (peaking at 5.0 % reflectance). We note that NIST measurements of the specular reflectance from this sample indicate a strong peak, approximately 2.5 % reflectance, in this region, and very little specular reflectance outside of this region, which may partially explain this discrepancy. At longer wavelengths (> 5.7 µm), our spectra compare well with the NIST spectra, with a maximum difference of less than 0.4 % (10 % in relative terms) from 10 µm to 14 µm.

For a sample of D25C16 (Figure S2c), the agreement between our corrected reflectance spectra and the spectrum measured by NIST was again very good. Similar spectral features at 3.0 µm, 3.4 µm, 5.7 µm, 6.8 µm, and 10.0 µm were seen in both spectra. Our corrected reflectance values were consistently about 0.4 % (10 % in relative terms) higher, across the entire spectrum, than the NIST measured reflectance. Repeatability of our measurements for this sample was again very high (95 % confidence interval width ≈ 0.5 %).

For a sample of ESLI Velvet (Figure S2d), which is composed of aligned carbon needles, there were no prominent reflectance features to compare between our corrected reflectance spectra and the spectrum measured by NIST. As was the case for the preceding three reference materials, we measured a somewhat higher reflectance than NIST. This bias was exhibited

across the entire spectrum, and tended to increase in size with increasing wavelength from an offset of about 0.1 % at 2 µm to 0.3 % at 7 µm (up to 40 % in relative terms).

To put the above comparisons in context, we note that the expanded uncertainty on the NIST measurements is estimated to be about 2.8 % (mean across 2 µm to 14 µm) for the NIST roughened gold sample (a high-reflectance reference material), and about 0.1 % (mean across 2 µm to 14 µm) for the ESLI Velvet sample (a low-reflectance reference material). We conduct a more formal uncertainty analysis below.

Finally, we conducted an analysis leveraging the overlapping spectral range (from 2.0 µm to 2.5 µm) of our two instruments, the PerkinElmer Lambda 750s (full range, 0.25 µm to 2.5 µm) and Nicolet iS10 (full range, 2.0 µm to 20 µm). Within this zone of overlap, there is a prominent peak in leaf reflectance at 2.2 µm. Despite differences in measurement technology, illumination sources, reflectance standards, and the fact that across this range both instruments are at the extreme edges of their spectral sensitivity, our analyses show that for two samples of varying reflectance (red maple leaves collected May 1 and June 26, respectively), the measured spectra are highly consistent between the two instruments (Figure S3a). Indeed, a comparison between 2.2 µm reflectance measured with the PerkinElmer instrument and 2.2 µm reflectance measured with the Nicolet instrument shows minimal bias and very high correlation (Figure S3b). In this comparison, we averaged the n = 3 samples collected for different species and crown positions; the error bars denote the standard deviation across these three replicates. This analysis gives us further confidence, not only in the spectral patterns but also in the overall magnitude of reflectance measured with the FTIR (Nicolet iS10) instrument.

Determination of expanded uncertainty

To quantitatively assess the uncertainties in our FTIR measurements, we followed the general approach described by Blake *et al.* (2018). We used the repeated measurements of three our standard materials, spanning a range of reflectance from highly reflective (roughened gold reference standard; Figure S2a) to minimally reflective (D25C16 and ESLI velvet; Figures S2c and d, respectively) to quantify random measurement uncertainties. We used the difference between the mean of our measurements and the NIST measurement of the same material to quantify systematic uncertainties. We included the uncertainty in the NIST measurement of the same material as an additional source of uncertainty. We then combined these three uncertainties in quadrature and multiplied by a coverage factor of k = 2 to obtain the expanded uncertainty. We report here the mean expanded uncertainty, calculated from 2 µm to 14 µm. These results are presented in Table S2. For all three standard materials, the systematic error was substantially larger than the random error. For the high-reflectance roughened gold reference standard, our expanded uncertainty was 3.83 %. For the two low-reflectance standard materials, D25C16 and ESLI velvet, our expanded uncertainties were 0.60 % and 0.51 %, respectively.

We then compared D25C16 and ESLI velvet expanded uncertainties, and their components, to the variability across replicate samples, and in relation to observed changes in reflectance over

time. The purpose of this analysis was to assess the relative magnitudes of biological variability and developmental change in relation to the overall uncertainty of our measurement system for materials that are superficially similar in reflectance to tree leaves. These results are shown in Table S3. We defined “biological variability” as the standard deviation across the n = 3 replicate samples collected for each species x crown position x sampling date combination. For the five species studied here, the mean biological variability (1 standard deviation) ranged from 0.39 % to 0.58 %, which is three- to four-fold larger than the random uncertainty (1 standard deviation) estimated for the low-reflectance standard materials, and comparable in magnitude to the expanded uncertainty for both D25C16 and ESLI velvet. We defined “developmental change” as the standard deviation across the mean reflectance measured on each of the different sun leaf sampling dates (n = 7 dates for red maple, paper birch, and trembling aspen; n = 6 dates for American beech; n = 5 dates for red oak). For the five tree species studied here, the developmental change (1 standard deviation) ranged from 3.2 % to 4.2 %, and is thus almost an order of magnitude larger than the biological variability across replicate samples.

Conclusion

Taken together, the above results give encouraging evidence for the robust repeatability of reflectance measurements with our setup, and the ability of our setup to measure reflectance of diffuse low-reflectance (< 10 % reflectance) samples that are in good agreement (in the context of random and systematic uncertainties) with measurement of the same sample at NIST. These findings are also consistent with results of our Nicolet vs. PerkinElmer comparison. The major difference between the spectra we measured and as measured by NIST appears, for the most part, to be a small upward bias in our spectra, even after correcting for overfilling of the sample port. With additional correction, this bias could be minimized or even eliminated.

Methods S2 Determination of diffuse and specular reflectance components.

Wong & Blevin (1967) concluded that "Leaves should not be regarded as perfectly specular or diffuse reflectors in the infrared region" but that there was a tendency for the specular component to increase with increasing wavelength. Salisbury (1986) reported that some leaf reflectance features in the MIR were strongly specular. But few, if any, studies have shown the full spectra for the diffuse and specular components of MIR leaf reflectance.

The Pike IntegratIR integrating sphere we used for 2.0 μm to 20 μm reflectance measurements features a specular exclusion port which—in principle—permits separation of total reflectance to its diffuse and specular components (Hanssen & Snail, 2001). However, we identified several design limitations that required modification of standard techniques in order to obtain satisfactory results. We describe these here.

The specular exclusion port is located on the underside of the sphere. The port cover slides between the open and closed positions by means of a knob on the front of the sphere. When the port is opened, the specular component from the sample is directed out of the sphere into a low-reflectance external cavity. Thus, when the port cover is opened, only the diffuse component remains in the sphere to be measured by the detector. The standard coating on the external cavity of the IntegratIR sphere is candle soot (email from Jeff Kuehl, Pike Technologies, 28 April 2017), which has long been used as a high-absorptance material in thermal radiation studies.

We conducted test measurements using a first surface mirror (6 mm thickness, Mirotek First Surface Mirrors, Rancho Dominguez, CA USA) on the open sample port. A first surface mirror should have very high specular reflectance and very low diffuse reflectance. We measured 94 % total reflectance from the first surface mirror. However, our diffuse reflectance measurement (i.e. with the specular exclusion port open) revealed reflectance artifacts that were wavelength-dependent and that could not be attributed to the optical properties of the mirror itself. Notably, our measurements indicated pronounced reflectance features across the range from 2 μm to 8 μm (Figure S4), which presumably arise from the spectral properties of the candle soot coating on the external cavity, and the resulting signal that is thus reflected back into the sphere. To reduce the reflectance from the external cavity we used a piece of ESLI Velvet (see reflectance spectra in Figure S2d) to line the external cavity. This resulted in a much lower (< 1 %), and generally featureless, diffuse reflectance spectrum for the first surface mirror. This measurement represents the aggregate of the actual diffuse component from the first surface mirror (which is expected to be negligible), plus any proportion of the specular component that is reflected back from the external cavity. The latter signal will be very small for samples with low total (and hence low specular) reflectance (e.g., leaves).

An additional limitation of the IntegratIR sphere design is that with the internal flipper mirror in reference position, the illuminating beam is directed at the exclusion port. This precludes standard comparison method measurements with the exclusion port open, because the

reference beam will be directed out of the port and into the external cavity, rather than at the wall of the sphere. An alternative, which we used here, is the method proposed by Blake *et al.* (2018), in which an intermediate point on the sphere wall is used for reference measurements. We chose to reference off the sphere wall with the flipper mirror positioned three-quarters of the way between sample and reference positions, i.e., midway between the illumination entrance port and the specular exclusion port. We observed that the reference measurements (V_{rs}, in Table S1) tended to be slightly higher, and thus measured sample reflectance slightly lower (by about 16.5 % in relative terms), when reference measurements were conducted with the mirror in the three-quarters position (note that this comparison is possible only with the specular exclusion port closed). Without correcting for this difference, we found that the measured reflectance of our Aeroglaze Z306 sample (see Methods S1) was biased low by 0.58 ± 0.07 % (mean ± 1 standard deviation, for wavelengths from 2 µm to 14 µm). Correcting for the difference in V_{rs} reduced the bias substantially. The corrected reflectance was biased low by only 0.05 ± 0.07 % (mean ± 1 standard deviation, for wavelengths from 2 µm to 14 µm), relative to the spectrum obtained when the reference measurement was made with the flipper mirror in the standard reference position.

Notes S1 Differences between sun and shade leaves.

Shade leaves tended to be larger in area (Figure 1, top), lower in both LMA_{DM} and LMA_{H2O} (Figure 1, second row), darker in color (Figure 1, third row), but similar in relative leaf color (Figure 1, fourth row) compared to the sun leaves collected on the same date. UV-VIS-NIR reflectance spectra were not markedly different between sun and shade leaves, although for some species there were regions of statistically significant differences, particularly in the NIR: shade leaf reflectance tended to be lower than sun leaf reflectance from 750 nm to 1300 nm, but the reverse was true from 1400 nm to 2500 nm (Figure S5). Within the MIR, the reflectance spectra of red maple, paper birch, and trembling aspen were very similar between sun and shade leaves (Figure S5b, S5d, and S5f), whereas there were obvious, and statistically significant, differences between sun and shade leaves of American beech and red oak (Figure S5h and S5j). Most notably, in these two species, shade leaves had pronounced reflectance peaks at 4 µm and 5.5 µm, and also at 10 µm, which were not observed for sun leaves. For all five species, Chl NDI and emissivity were extremely similar between sun and shade leaves (Figure 4).

Notes S2 Diffuse and specular components of total reflectance.

For all species, total reflectance was largely dominated by diffuse reflectance, with the specular component accounting for a much smaller fraction (Table S4). In general, the mean specular reflectance of shade leaves was similar to that of sun leaves, and the specular component accounted for only ≈ 10 % of total reflectance. The one exception to this pattern was red oak sun leaves, which were noticeably glabrous and for which the specular component accounted for almost 20 % of total reflectance, compared to less than 10 % of total reflectance for shade leaves of the same species.

Across all wavelengths, specular reflectance was extremely low, generally 1 % or less (Figure S6). For all species, there was a bump in the specular component at 2.2 µm, but even then specular reflectance did not exceed 2 %, whereas diffuse reflectance at the same wavelengths approached 20 %. In general, pronounced spectral features were difficult to discern in the specular spectra, given the extremely small specular signal. However, for at least paper birch, red oak, and American beech, the spectral peaks at 3.4 µm and 3.5 µm that were identified in the total reflectance spectra (Figure 2), could also be identified in both the diffuse and specular reflectance spectra.

The specular reflectance spectra of shade leaves were generally similar to those of sun leaves, with the exception of red oak (compare Figure S6i and S6j). For red oak sun leaves, there was a rising trend in both diffuse and specular reflectance above 10 µm, but this was not observed in red oak shade leaves.

References for Supplementary Material

Blake TA, Johnson TJ, Tonkyn RG, Forland BM, Myers TL, Brauer CS, Su Y-F, Bernacki BE, Hanssen L, Gonzalez G. **2018**. Methods for quantitative infrared directional-hemispherical and diffuse reflectance measurements using an FTIR and a commercial integrating sphere. *Applied Optics* **57**: 432.

Elvidge CD. **1988**. Thermal infrared reflectance of dry plant materials: 2.5-20.0 µm. *Remote Sensing of Environment* **26**: 265–285.

Hanssen LM, Snail KA. **2001**. Integrating spheres for mid- and near-infrared reflection spectroscopy. In: Griffiths PR, ed. Handbook of Vibrational Spectroscopy. Chichester, UK: John Wiley & Sons, Ltd.

Hecker C, Hook S, Meijde M van der, Bakker W, Werff H van der, Wilbrink H, Ruitenbeek F van, Smeth B de, Meer F van der. **2011**. Thermal infrared spectrometer for earth science remote sensing applications—Instrument modifications and measurement procedures. *Sensors* **11**: 10981–10999.

Salisbury JW. **1986**. Preliminary measurements of leaf spectral reflectance in the 8-14 micrometer region. *International Journal of Remote Sensing* **7**: 1879–1886.

Wong C, Blevin W. **1967**. Infrared reflectances of plant leaves. *Australian Journal of Biological Sciences* **20**: 501–508.
